# Neuromuscular effects of repeated exposure to the pesticide methyl bromide

**DOI:** 10.1371/journal.pone.0357385

**Published:** 2026-08-31

**Authors:** Dakota R. McMeans, Hannah S. Davidson, Martha J. Sonner, Shannon H. Romer

**Affiliations:** 1 Naval Medical Research Unit Dayton, Environmental Health Effects Laboratory, Wright-Patterson A.F.B., Dayton, Ohio, United States of America; 2 Leidos Inc, Reston, Virginia, United States of America; 3 Department of Neuroscience, Cell Biology, & Physiology, Wright State University, Dayton, Ohio, United States of America; 4 Oak Ridge Institute of Science and Education (ORISE), Oak Ridge, Tennessee, United States of America; Fujita Health University, JAPAN

## Abstract

Methyl bromide (MB) is a fumigant pesticide that remains in use for quarantine and pre-shipment applications. Although acute intoxication is associated with motor dysfunction, the effects of repeated occupational inhalation exposure on neuromuscular structures remain poorly characterized. This study evaluated functional and anatomical consequences of repeated MB inhalation exposure in adult male Sprague–Dawley rats. Animals were exposed to either clean air or 300 ppm MB by nose-only inhalation for 2 hours a day, 5 days a week, over 4 weeks. Neurobehavioral assessments included functional observation battery testing, gait analysis, rotarod performance, and open-field locomotor activity. Lumbar spinal cord and hindlimb skeletal muscles were analyzed using quantitative confocal microscopy to assess motoneuron soma morphology, astrocyte reactivity, and neuromuscular junction (NMJ) structure. Repeated MB exposure did not produce overt clinical toxicity, body weight loss, or measurable impairments in gait, motor coordination, or spontaneous locomotor activity. No differences in lumbar motoneuron number or spinal astrocyte reactivity were detected between groups. However, MB exposure selectively reduced soma cross-sectional areas of the largest lumbar motoneurons. In parallel, NMJs within the predominantly fast-twitch extensor digitorum longus muscle exhibited increased denervation and decreased postsynaptic nicotinic acetylcholine receptor occupancy relative to controls. These alterations were not observed in the predominantly slow-twitch soleus muscle. These findings demonstrate that repeated subacute MB exposure can induce subtle peripheral neuromuscular pathology in the absence of overt behavioral dysfunction. Furthermore, these findings suggest that distal motor terminals and associated synaptic structures may represent selective and early targets of MB neurotoxicity. Collectively, the results support the concept that repeated occupational-like MB exposure may produce subclinical peripheral motor system injury prior to the onset of detectable functional impairment.

## Background

Methyl bromide (MB) is a colorless, odorless fumigant historically used worldwide for pest control in agriculture, quarantine treatment of imported goods, and structural fumigation. Although global use has declined following international restrictions under the Montreal Protocol, MB continues to be used for quarantine and pre-shipment fumigation and remains a potential occupational hazard in agricultural, shipping, and port environments. Human poisoning cases consistently identify the nervous system as a major target of toxicity with prominent motor abnormalities including muscle weakness, impaired coordination, and gait disturbance [1–3]. Subacute and repeated exposures at lower concentrations are also reported in occupational settings and may produce similar motor deficits accompanied by sensory disturbances such as paresthesia and reduced cutaneous sensation [2,4,5].

The mechanisms underlying MB neurotoxicity remain incompletely understood, although several biochemical pathways have been proposed. Experimental studies in rodents indicate that inhaled MB distributes broadly throughout the body without preferential accumulation in the central nervous system [6–8]. MB is a highly reactive alkylating agent that undergoes nucleophilic substitution reactions with cellular molecules including glutathione, proteins, and nucleic acids. These reactions can lead to depletion of intracellular glutathione, disruption of redox homeostasis, and downstream oxidative stress [9–12]. Additionally, MB exposure has also been proposed to interfere with cholinergic signaling. Aberrant methylation reactions may generate methyl phosphate intermediates capable of inhibiting acetylcholinesterase (AChE), potentially leading to cholinergic overstimulation and associated neuromotor symptoms [9]. Clinical observations following repeated low-level MB exposure, including tremor, salivation, nausea, and vomiting, are consistent with cholinergic dysfunction and may reflect partial inhibition of cholinesterase activity [13,14]. Although the relative contribution of these mechanisms remains uncertain, MB neurotoxicity likely arises from a combination of alkylation-induced oxidative stress, disruption of cellular metabolism, and interference with cholinergic neurotransmission.

Motor dysfunction following MB exposure has traditionally been attributed to injury within the central nervous system, particularly the cerebellum. Multiple clinical reports describe cerebellar signs such as gait ataxia, dysarthria, and impaired coordination in poisoned patients. These clinical deficits likely reflect underlying structural damage, as neuroimaging studies have identified symmetric lesions in cerebellar and brainstem structures following acute intoxication [2,15]. However, increasing evidence indicates that MB neurotoxicity is not restricted to central structures. Clinical case reports and electrophysiological studies demonstrate axonal peripheral axonopathy following exposure, suggesting that peripheral motor components may also contribute to motor impairment. For example, acute MB poisoning has been associated with progressive limb weakness and nerve-conduction alterations, consistent with axonal neuropathy [16,17]. These findings indicate that peripheral nerve injury may occur alongside central neurological damage.

Evidence from occupational exposure scenarios further supports a peripheral component to MB neurotoxicity. Workers exposed to MB fumigation have developed lower-limb neuropathy, gait instability, sensory deficits, and persistent neurological sequelae, consistent with a distal axonopathy or a “dying-back” neuropathic pattern [18]. Consistent with these clinical observations, acute inhalation exposure in rats reduced locomotor activity at concentrations ≥188 ppm, while repeated exposures of 200–300 ppm for several weeks produced persistent alterations in spontaneous activity and peripheral nerve function [19]. Lower chronic exposure concentrations have produced more variable effects, but still include neuropathic changes, such as reduced nerve conduction velocity in rabbits following repeated MB inhalation [20]. Together, these findings demonstrate that MB exposure can produce locomotor impairment and peripheral nerve dysfunction in both humans and experimental animals.

Occupational exposure limits for MB were established largely to prevent acute neurotoxicity observed in exposed workers and laboratory animals. The National Institute for Occupational Safety and Health reports rat inhalation LC₅₀ values of approximately 2,833 ppm for a 30-min exposure and 755 ppm for an 8-hour exposure and established an Immediately Dangerous to Life or Health (IDLH) concentration of 250 ppm for MB [21]. In the United States, the Occupational Safety and Health Administration sets a permissible exposure limit ceiling of 20 ppm for workplace exposure [22], while the American Conference of Governmental Industrial Hygienists recommends a more protective threshold limit value of 1 ppm per 8 hour workday within a 40 hour work week for a working lifetime [23]. Collectively, these exposure limits underscore the neurotoxic potential of MB and provide a regulatory framework for evaluating the health risks associated with inhalational exposure.

Despite international restrictions, MB continues to be used for quarantine fumigation of cargo and agricultural products, including in ports and shipping environments. Naval forces frequently interact with commercial shipping and port infrastructure where fumigants such as MB are used for pest control of cargo and storage facilities. As a result, Service Members may be exposed to MB during port operations, logistics support activities, and inspection of fumigated cargo. Environmental persistence of MB following application may further increase the potential for incidental exposure in operational settings [24–26]. Because motor coordination and neuromuscular performance are essential for operational readiness and mission performance, understanding the neurological consequences of repeated MB exposure is of particular relevance for military occupational health, as well as workers within the general public.

Although previous studies have largely focused on cerebellar pathology and central nervous system injury, the potential contribution of peripheral motor structures to MB-induced motor dysfunction has not been well characterized. In particular, the effects of MB exposure on spinal motoneurons, neuromuscular junction morphology, and skeletal muscle denervation remain poorly understood. This study therefore aimed to evaluate anatomical and functional impacts of repeated MB exposure. Adult male Sprague–Dawley rats were exposed to MB using a controlled nose-only inhalation exposure system to examine central and peripheral motor system outcomes following repeated exposure at a concentration that represents a sublethal exposure scenario.

## Methods

The primary objective of this study was to identify functional and anatomical changes from MB exposure in a rat model. Animals were exposed to 300 ppm MB gas diluted in clean air or to clean air. Our technical approach used a combination of *in vivo* neurobehavioral studies and quantitative confocal microscopy to assess exposure effects. All procedures were conducted in accordance with Wright-Patterson Institutional Animal Care and Use Committee (IACUC) guidelines and adhered to ARRIVE reporting recommendations.

### Animal subjects

Research was conducted under an approved animal use protocol in an AAALAC International-accredited facility in compliance with all federal statutes and regulations relating to animals and experiments involving animals and adheres to principles stated in the Guide for the Care and Use of Laboratory Animals, NRC Publication, 2011 edition. A total of 18 young adult male Sprague Dawley rats (n = 9 per group) were used. Animals were housed under a 12 h light/dark cycle with *ad libitum* access to standard chow and water in a temperature and humidity controlled vivarium. Animals were observed at least twice daily by trained personnel for general health and for signs of pain, distress, or illness. Humane endpoints were predefined and included >20% body weight loss, persistent hypoactivity or inability to access food or water, labored breathing, or any condition considered moribund by the attending veterinarian. Animals meeting these criteria would be promptly removed from the study and humanely euthanized. No animals reached these humane endpoints, exhibited unrelieved pain or distress, or died prior to the planned study endpoint. At study completion, animals were euthanized in accordance with the American Veterinary Medical Association Guidelines for the Euthanasia of Animals. Euthanasia was performed via exsanguination during transcardial perfusion under deep anesthesia induced by sodium pentobarbital (150 mg/kg body weight, intraperitoneal) with all efforts made to minimize pain and distress (please see “Transcardial perfusions and tissue preparation”).

### Exposures and experimental timeline

Animals were repeatedly exposed to either methyl bromide (MB) via inhalation at 300 parts per million (ppm) or clean air control for two hours per day, five days per week, for a total of four weeks. This concentration was selected based on previous experimental studies investigating MB neurotoxicity and behavioral effects in rodents [20,27–30]. The exposure paradigm was designed to model repeated occupational-like inhalation exposure without inducing mortality and is consistent with the 28-day framework for subacute inhalation toxicity described in Organisation for Economic Co-operation and Development Test Guideline 412 [31], and remained below concentrations associated with short-duration lethality in rats. MB was delivered as compressed liquefied gas (37.4% methyl bromide by volume in air) obtained from SynQuest Laboratories (Alachua, FL).

The total experimental duration was six weeks. The pre-exposure week was allocated for acclimation to the inhalation exposure system. This was followed by four weeks of daily exposure. The final week post-exposure was dedicated to neurobehavioral testing and study completion. Animal cohorts were staggered at study initiation, such that half of both the control and MB-exposed groups commenced exposure one week later than the remaining animals.

### Nose-only inhalation exposure system

Methyl bromide (MB) inhalation exposures were conducted using a multi-port nose-only exposure system (NOES) consisting of a 52-port nose-only exposure unit (NOEU; Lab Products, Seaford, DE). Animals were placed in individual open-end restraint tubes connected to the exposure manifold, allowing delivery of test atmospheres directly to the breathing zone. The NOEU operated as a push–pull system in which a positive supply airflow delivered test atmosphere through the inner plenum and a negative exhaust removed excess atmosphere and exhaled air through the outer plenum. Unused ports were sealed with solid rods to maintain consistent airflow distribution. Two independent NOEUs were used concurrently for each experimental group (clean air control and 300 ppm MB). Each exposure unit was housed in a separate negative-pressure ventilated hood to prevent cross-contamination between exposure groups and to protect laboratory personnel.

To reduce restraint-induced stress during inhalation exposures, all animals were acclimated to the NOEU restraint tubes during the week preceding the exposure period. Animals underwent one acclimation session per day for five consecutive days, with restraint durations progressively increased from 5 to 30, 60, and 120 minutes. This graduated acclimation protocol familiarized the animals with the restraint apparatus before study initiation and minimized nonspecific stress responses associated with the exposure procedure. Animals assigned to both the MB exposure and control groups underwent the identical acclimation protocol to ensure that any nonspecific effects of restraint or the exposure system were balanced across treatment groups.

### Exposure airflow control and environmental conditions

MB exposure atmospheres were generated by proportionally mixing compressed MB gas with filtered house compressed air. MB was supplied from a compressed gas cylinder (37.4% MB by volume in air; SynQuest Laboratories, Alachua, FL) regulated to approximately 15 psig using a dual-stage regulator (Matheson Tri-Gas, Dayton, OH). The MB-air mixture was introduced into the inlet of each NOEU, where it was further mixed with dilution air before entering the exposure manifold. Dilution air flows were controlled using rotameters to maintain a constant total airflow and achieve target exposure concentrations. Test atmospheres were delivered at a total flow rate of approximately 13 L min^−1^ through the central plenum of each NOEU, providing approximately 0.5 L min^−1^ of fresh test atmosphere to each open exposure port. Exhaust flow was adjusted to maintain chamber static pressure between 0.00 and −0.20 inches H_2_O (target −0.05 inches) for MB exposures and between 0.00 to 0.20 inches H_2_O (target 0.05 inches) for control chambers. These operating conditions maintained a stable exposure atmosphere and continuous replenishment of the breathing zone throughout each exposure. The airflow delivered to each exposure port exceeded the resting minute ventilation of an adult rat, thereby minimizing the potential for rebreathing of exhaled gases.

Environmental conditions were continuously monitored throughout exposures. Temperature and relative humidity were measured using a humidity and temperature probe (Rotronic model HF532WB6XD1XX with HC2-S probe; Rotronic Instruments Inc., Hauppauge, NY), and static pressure was measured using a differential pressure sensor (ZPS-05-SR09-EZ-ST-D; Building Automation Products Inc., Gays Mills, WI). Sensors were positioned at representative exposure ports within each NOEU. Environmental data were recorded once per second using a data acquisition system (NI-9207 module within a NI cDAQ-9178 CompactDAQ chassis; National Instruments, Austin, TX) and logged using LabVIEW software (v12.0; National Instruments).

### Analytical verification of exposure concentration

MB concentrations in the exposure atmospheres were measured using Fourier Transform Infrared Spectroscopy (FTIR) (Thermo Scientific Nicolet iS10, Thermo Fisher Scientific, Waltham, MA) equipped with a 2-meter gas cell, which provided appropriate sensitivity for mid-ppm concentration measurements. Spectral acquisition, processing, and reporting were automated using a custom macro written in OMNIC™ software, producing measurements at approximately 22-second intervals.

Quantification was performed using characteristic MB absorbance peaks at 2983.2 cm ^−1^ and 2959.0 cm^−1^. Instrument calibration was conducted prior to exposures using MB standards generated in 100-L Tedlar gas bags (SKC Inc., Covington, GA). Calibration atmospheres were prepared by diluting certified MB gas (99.9% purity; SynQuest Laboratories) into compressed air using gas-tight syringes to produce concentrations of 50, 150, 225, 300, and 400 ppm.

Calibration curves were generated according to the Beer–Lambert relationship, producing linear responses across the tested concentration range with R² > 0.999. These calibration equations were incorporated into the FTIR macro to enable real-time monitoring of exposure concentrations during experiments, ensuring both animal safety and accurate maintenance of target concentrations.

### Neurobehavior

#### Functional observation battery.

A Functional Observation Battery (FOB) was used to assess the general health, neurological function, and overall wellbeing of rats three days following the final exposure. The FOB consists of a standardized series of qualitative and quantitative clinical observations designed to detect gross functional changes in physical condition, sensorimotor responses, and behavioral performance. Each animal was observed in three contexts. First, within the home cage to evaluate spontaneous activity and posture. Second, immediately upon removal from the cage to assess reactivity, vocalization, and handling responses. Finally, during manual restraint to evaluate muscle tone, reflexes, and autonomic signs. All assessments were conducted by trained personnel under controlled environmental conditions to minimize confounding stimuli, including consistent temperature, humidity, lighting, and noise levels, and the absence of distracting odors. This approach ensured that any observed changes could be attributed to exposure-related effects rather than external environmental influences.

### Quantitative gait analysis

Gait analysis was performed using a Kinetic Weight Bearing system (Bioseb, Vitrolles, France) six days following the final exposure. Rats traversed a walled corridor (130 cm length × 18 cm height) equipped with a pressure-sensitive sensor mat containing approximately 6,000 individual sensors. The system independently tracked each paw, and pressure data were acquired and processed using the manufacturer’s proprietary software. Synchronized video recordings were used to monitor forward progression and to verify paw placement for gait quantification. The software provided measures of applied force, weight distribution, speed, and acceleration for each paw. A trial was considered successful when the rat traversed at least three-fifths of the corridor within 10 seconds. Approximately three successful trials were collected per animal. For the purposes of this study, data from individual forepaws and hindpaws were grouped for analysis.

### Accelerod

The accelerod assay was conducted using a rotarod system (AccuRotor EzRod, Omnitech Electronics, Inc., Columbus, OH, USA) equipped with a modified cover to accommodate larger rats (>300 g). Animals were first trained to a minimum performance criterion of 11 rotations per minute (RPM) for 5 minutes on days four and five following the final exposure. To maintain naïveté to acceleration, speed ramping was not used during training. For the accelerod test performed six days following the final exposure, the rotarod was programmed to accelerate from 4 to 40 rpm over 300 seconds. The test commenced at the onset of acceleration and concluded when the rat either fell from the rod or completed the full 300-second duration. A trial was considered valid if the animal remained on the rod for at least 60 seconds. Total distance traveled, latency to fall, and maximum speed were recorded.

### Open field motor activity

Open-field motor activity was assessed three days following the final exposure using an automated Photobeam Activity System (PAS; San Diego Instruments, San Diego, CA, USA) in a 16 × 16 in polycarbonate arena. Horizontal and vertical photobeams detected ambulatory and rearing movements while rats freely explored the open field. Locomotor activity was defined as sequential interruption of two adjacent photobeams, whereas fine movements were defined as single-beam interruptions. Activity was recorded and quantified using the PAS software (San Diego Instruments). Each rat was monitored for 30 minutes under controlled environmental conditions, including standardized lighting, temperature, humidity, and minimal background noise and odors. Behavioral endpoints quantified included total distance traveled, active time, locomotor speed, number of vertical rears, fine movements, and the percentage of time spent in the center versus peripheral regions of the arena.

### Immunohistochemistry and microscopy

#### Transcardial perfusion and tissue preparation.

Seven days following the final exposure, rats were deeply anesthetized with sodium pentobarbital (150 mg kg^−1^, intraperitoneal). Adequate anesthetic depth was confirmed by absence of reflex responses to noxious stimuli prior to proceeding, in accordance with the American Veterinary Medical Association Guidelines for the Euthanasia of Animals. Euthanasia was achieved by exsanguination during transcardial perfusion under deep anesthesia. Briefly, the thoracic cavity was opened, a perfusion cannula was inserted into the left ventricle, and the right atrium was incised to permit drainage. Using a peristaltic pump, the vasculature was initially flushed with an isotonic physiological buffer to remove blood, followed by fixation with 4% paraformaldehyde (PFA) in 0.1 M phosphate buffer (pH 7.3). Following perfusion and euthanasia, spinal cords and skeletal muscles were rapidly dissected and post-fixed in 4% PFA for 1–2 h at room temperature prior to further processing. All procedures were performed in a manner designed to minimize pain, distress, and tissue degradation.

### Spinal cord immunohistochemistry and microscopic imaging

Following fixation, spinal cord tissues were cryoprotected in 15% sucrose prepared in 0.1 M phosphate buffer at 4 °C until sectioning. Spinal cord tissue was sectioned at 50 µm thickness using a freezing sliding microtome (SM2010R, Leica Biosystems) equipped with a freezing stage (BFS-40MP, Physitemp Instruments, Clifton, NJ, USA). Sections were collected in 0.01 M phosphate-buffered saline (PBS; pH 7.4). Tissue sections were blocked for 45 min at room temperature in normal horse serum (10%) prepared in PBS containing 0.1% Triton X-100. Primary antibodies were applied overnight at 4 °C. Antibodies included chicken anti-GFAP (Invitrogen PA1–10004; 1:1000, Carlsbad, CA, USA) to label astrocytes. Neuronal cell bodies were labeled using Nissl Green (Invitrogen N21480; 1:100, Carlsbad, CA, USA). Following primary antibody incubation, sections were washed in PBS and incubated with species-appropriate fluorophore-conjugated secondary antibodies, including donkey anti-mouse (Jackson ImmunoResearch 715-605-151; 1:100, West Grove, PA, USA) and donkey anti-chicken (Jackson ImmunoResearch 703-165-155; 1:100, West Grove, PA, USA). Sections were mounted on glass microscope slides and coverslipped using antifade mounting medium (Vectashield; Vector Laboratories H-1000, Newark, CA, USA).

Confocal micrographs were acquired using a Leica SP8 confocal microscope (Leica Microsystems, Deerfield, IL, USA) equipped with a 20 × objective. Z-stack images were collected at 1 µm intervals across a depth of 10 µm. Image acquisition parameters were kept constant across experimental groups. All image analyses were conducted on original unmodified images by investigators blinded to treatment group.

### Neuromuscular junction preparations and microscopic imaging

The extensor digitorum longus (EDL) and soleus muscles were dissected bilaterally following perfusion and euthanasia. Muscles were flattened between glass slides and fixed in 4% PFA for 1 hour, then transferred to PBS for storage. Whole muscles were blocked in 10% normal horse serum prepared in PBS containing 0.3% Triton X-100 for 90 min at room temperature. Muscles were then incubated overnight at 4 °C in PBS containing 0.3% Triton X-100 with the following primary antibodies: rabbit anti-SV2B (Cedarlane Laboratories 11902SY; 1:2000) to label synaptic vesicles and mouse anti-neurofilament-heavy chain (NF-H; BioLegend 801601; 1:500) to label axons. Following washes in PBS-Triton X-100 (0.3%), muscles were incubated for 2–4 hours with fluorescent secondary reagent donkey anti-rabbit Cy3 (Jackson ImmunoResearch 711-605-152; 1:100, West Grove, PA, USA) and donkey anti-mouse Alexa Fluor 647 (Jackson ImmunoResearch 715-605-151; 1:100, West Grove, PA, USA) combined with α-bungarotoxin Alexa Fluor 488 (Molecular Probes B13422; 1:1000) to label postsynaptic nicotinic acetylcholine receptors (nAchR). Whole muscles were mounted in glass imaging chambers and submerged in glycerol mounting medium prior to imaging.

Neuromuscular junctions (NMJs) were imaged using a Leica SP8 confocal microscope with a 20 × objective lens. Z-stack images were acquired at 1 µm intervals across the NMJ band. Large fields containing ≥20 NMJs were captured from each muscle for analysis. NMJ innervation status was evaluated using flattened confocal projections in Leica Application Suite X software. Each NMJ was categorized into one of three groups. (1) Fully innervated: presynaptic NF-H labeling overlapped most or all of the postsynaptic nAchR signal. (2) Partially denervated: presynaptic NF-H labeling covered approximately half of the nAchR signal. (3) Fully denervated: presynaptic NF-H labeling was absent or nearly absent from the postsynaptic receptor field. The frequency of each innervation category was calculated for each muscle and averaged across animals within each experimental group.

The synaptic morphology of the NMJ was assessed with several metrics, including NMJ area, nAchR Area, nAchR fragmentation, nAchR pixel heterogeneity, nerve terminal Area, and nerve terminal pixel heterogeneity. The NMJ area was defined as the entire area of the outermost ellipsoid shape containing all nAchR signal of a single NMJ. The nAchR area is the absolute area of the nAchR associated signal of a single NMJ. The nAchR fragment count was assessed based on the discrete count of unique and continuous isolated regions of nAchR. The pixel heterogeneity is a measure of signal intensity variability, ranging from 0 to 1, with 0 representing a completely homogenous signal intensity and 1 representing a maximally heterogenous signal intensity. Pixel heterogeneity was assessed from regions of the image expressing fluorescent signal associated to nAchRs. The nerve terminal metrics were assessed in the same fashion as the nAchR metrics, except for using the signal detected from SV2B and NFH immunoreactivity within each NMJ area. Several derived metrics were also calculated from these primary measurements, such as nAchR occupancy (nAchR area / NMJ area), nerve terminal occupancy (nerve terminal area / NMJ area), and nerve terminal area / nAchR area.

### Statistical approaches

All testing and analyses were performed by investigators blinded to exposure conditions. For all statistical tests, the level of significance was set at p < 0.05. Unless otherwise stated, statistics are presented as mean ± standard deviation in text and tables and mean ± standard error of the mean in figures. All analyses were performed using SigmaPlot software unless otherwise noted.

Unless otherwise specified, the animal was treated as the experimental unit, and statistical analyses were conducted using animal-level summary values. Prior to group comparisons, data were evaluated for normality using the Shapiro–Wilk test and for homogeneity of variance using Levene’s test. If both assumptions were satisfied, differences between two groups were assessed using an unpaired two-tailed Student’s t-test. If normality was satisfied but homogeneity of variance was not, Welch’s t-test was used. If normality was not satisfied in either group, a nonparametric Mann–Whitney U test was applied.

For motoneuron (MN) soma size, cross-sectional area (µm²) was measured at the individual neuron level, resulting in a hierarchical dataset with many neurons nested within animals. To compare soma cross-sectional area between control and MB-exposed groups while accounting for within-animal dependence, linear mixed-effects models were fitted with exposure group as a fixed effect and animal as a random intercept. The use of a mixed-effects model prevented pseudoreplication by treating animal as the experimental unit while accounting for the correlation among neurons sampled from the same animal. The primary model was fitted to the natural log-transformed soma area to improve adherence to assumptions of homoscedasticity and residual normality for this strictly positive morphometric outcome. Parameters were estimated using restricted maximum likelihood. For interpretability, fixed-effect estimates from the log-scale model were exponentiated and are reported as multiplicative percent differences between groups. Model adequacy was evaluated by inspection of residual distributions, including assessment of residual symmetry, normality, and extreme standardized residuals, as well as residual variance across exposure groups. The log-transformed model was retained as the primary analysis when it better satisfied model assumptions. As a sensitivity analysis, the same random-intercept structure was also fitted to the untransformed area data to assess robustness of the findings. Mixed-effects analyses were conducted in Python using statsmodels.

As for MNs, NMJ synaptic morphology metrics were also analyzed via mixed-effects modeling implemented in R (version 4.5.0). In all models, group, muscle, and their interaction (group*muscle) were defined as categorical fixed effects, with animal identity included as a random intercept. 460 NMJs were analyzed in total, unless assessing metrics related to presynaptic morphology, of which 403 NMJs that showed positive presynaptic staining were included. Area based metrics with data expressing a Gaussian distribution were fitted using a stand linear mixed model, using the *lme4* R package (*lmer* function). Pixel heterogeneity and relative area occupancy metrics were analyzed as beta regressions, due to these data being bound to a 0–1 range. Beta regression was modeled using the *glmmTMB* R package (*glmmTMB* function). The probability of presynaptic presence was handled as a binomial distribution using the *lme4* R packge (*glmer* function). Finally, nAchR fragment count was handled as a Poisson distribution using the *glmmTMB* R package (*glmmTMB* function). All models were evaluated for convergence, and none yielded singular fits. Q-Q plots of all models were also inspected, and log transformations were applied to handle heteroskedasticity when present. Post-hoc pairwise comparisons were computed using either a two-sided t-test or Wald z-test on estimated marginal means (computed via the *emmeans* R package), with a Tukey method adjustment to control for type I error rates across multiple comparisons.

For further nAchR area analysis, data were analyzed by comparing the nAchR area across similarly sized NMJs, using a linear mixed effects (*lme4* and *lmerTest* R packages). The nAchR area was modeled as a function of NMJ area, experimental group, and muscle type. Animal identity was included as a random intercept to account for the hierarchical structure of the dataset. NMJ area was mean-centered prior to analysis to improve interpretability, allowing comparisons of nAchR area across groups at equivalent NMJ sizes. The quality of fit was assessed by visual analysis of a residual Q-Q plot, which indicated approximate normality, with minor deviations at the tails. Furthermore, a residual vs. fitted plot demonstrated an increase of variance at the right tail of the distribution (confirmed by correlation between absolute residual and fitted values: r = 0.39), suggesting mild heteroskedasticity. Residuals of nAchR area showed no systematic relationship with NMJ area (r ≈ 0), supporting the linear model specification. To account for mild heteroskedasticity, a robust linear mixed model was run in parallel (using the *robustlmm* R package), which yielded consistent model estimates and did not alter the direction of significance of primary effects, supporting the stability of model inference.

## Results

### Health and clinical assessments following repeated MB exposure

Body weight was monitored weekly as a general indicator of animal health throughout the study (S1 Table in S1 File; S1 Fig, Dataset 1). Previous studies have reported decreased body weight following exposure to MB in rats [28,32]. In the present study, body weights increased progressively over time in both groups, and no differences were detected between control and 300 ppm MB-exposed animals at any time point. Body weight trajectories were consistent with normal growth for animals of this age.

Animal health and well-being were further evaluated through daily clinical observations on exposure days, and a detailed functional observation battery (FOB) conducted three days after the final exposure (Dataset 2). No overt clinical abnormalities were observed in any animals. All rats maintained normal posture and exhibited no tremor, spasm, or seizure activity. Behavior and handling reactivity were unremarkable, and no piloerection, vocalization, porphyrin staining, or changes in arousal were observed. Skin, gingival, and nasal appearance were normal, and respiration remained unlabored. Neurological and autonomic responses assessed during manual manipulation and restraint, including muscle tone, tail-pinch response, acoustic startle, and righting reflexes, were within expected physiological ranges in both groups. Forelimb and hindlimb grip strength did not differ between treatment groups (Table 1). Similarly, pressure-based nociceptive sensitivity measured using a small animal algometer (SMALGO) was unchanged (Table 1). Quantitative gait analysis also revealed no differences between groups (Table 2, S2 and S3 Figs). Together, these findings indicate that repeated MB exposure under the conditions tested did not produce detectable alterations in body weight, clinical observations, or functional neurological measures in rats.

**Table 1:** Grip strength and small animal algometer (SMALGO) outcomes.

Metric	Control				MB				p-value	Effect Size
	Mean	SD	SEM	n	Mean	SD	SEM	n		
Forelimb Grip Strength (KgF)	0.492	0.173	0.058	9	0.453	0.084	0.028	9	p = 0.549	d = 0.29
Hindlimb Grip Strength (KgF)	0.46	0.156	0.052	9	0.38	0.12	0.04	9	p = 0.237	d = 0.58
SMALGO (g)	406.4	150.2	50.1	9	347.1	156.7	52.2	9	p = 0.424	d = 0.38

Statistical comparisons between groups were performed using an independent two-sample t-test. n = number of animals sampled.

**Table 2:** Quantitative Gait Analysis.

Metric	Unit	Control				MB				p-value	Effect Size
		Mean	SD	SEM	n	Mean	SD	SEM	n		
Speed	cm/s	24.0	7.3	2.4	9	21.9	4.7	1.5	9	p = 0.471	d = 0.35
Forelimb Peak Force	cN	98.5	12.7	4.2	9	90.2	11.0	3.6	9	p = 0.160	d = 0.69
Hindlimb Peak Force	cN	109.5	18.2	6.0	9	111.5	22.8	7.6	9	p = 0.840	d = −0.09
Forelimb Peak Surface Contact	cm^2^	1.428	0.189	0.063	9	1.483	0.192	0.064	9	p = 0.544	d = −0.29
Hindlimb Peak Surface Contact	cm^2^	1.478	0.229	0.076	9	1.411	0.262	0.087	9	p = 0.573	d = 0.27
Forelimb Stride Length	cm	15.45	1.84	0.61	9	15.24	2.81	0.93	9	p = 0.853	d = 0.09
Hindlimb Stride Length	cm	17.16	3.79	1.26	9	16.08	3.18	1.06	9	p = 0.525	d = 0.31
Forelimb Swing Duration	ms	354.7	84.7	28.2	9	332.6	89.6	29.8	9	p = 0.596^†^	δ = 0.16
Hindlimb Swing Duration	ms	401.3	159.6	53.2	9	340.7	185.6	61.8	9	p = 0.426^†^	δ = 0.23
Forelimb Stance Duration	ms	319.7	88.1	29.3	9	322.9	63.8	21.2	9	p = 0.931	d = −0.04
Hindlimb Stance Duration	ms	361.7	116.4	38.8	9	369.8	98.8	32.9	9	p = 0.875	d = −0.08
Forelimb Propulsion Duration	ms	118.0	38.6	12.8	9	125.8	33.8	11.2	9	p = 0.651	d = −0.22
Hindlimb Propulsion Duration	ms	202.2	62.19	20.7	9	220.5	66.7	22.2	9	p = 0.556	d = −0.28
Forelimb Step Duration	ms	674.4	158.2	52.7	9	655.4	116.5	38.8	9	p = 0.775	d = 0.14
Hindlimb Step Duration	ms	762.9	238.5	79.5	9	710.3	230.8	76.9	9	p = 0.641	d = 0.22

Statistical comparisons between groups were performed using an independent two-sample t-test. (†) – Wilcoxon rank sum test. n = number of animals sampled.

### Motor coordination, balance, and open field activity following repeated MB exposure

Motor coordination and balance were evaluated using the accelerod assay (Dataset 2). Rats were trained to walk on a rotating rod (rotarod) at a constant speed over several days until proficient. Training occurred on days four and five following MB exposure, and the accelerod test was performed on day six post-exposure. All animals met the minimum performance criterion of maintaining balance at 11 rotations per minute (RPM) for 5 minutes prior to testing. During the test, the rod gradually increased in speed, requiring the animals to continually adjust their coordination and balance to remain on the rotating rod. Performance metrics included latency to fall, final rotational speed (RPM), and total distance traveled. No significant differences were observed between control and MB-exposed rats for rotarod performance measure (Table 3).

**Table 3:** Accelerod Results.

Metric	Unit	Control				MB				p-value	Effect Size
		Mean	SD	SEM	n	Mean	SD	SEM	n		
Test Duration	s	139.15	44.19	14.73	9	153.28	25.37	8.45	9	p = 0.417	d = −0.39
Final Speed	RPM	20.7	5.3	1.7	9	22.3	3.0	1.0	9	p = 0.418	d = −0.39
Distance Traveled	cm	668.1	342.0	114.0	9	754.1	214.5	71.5	9	p = 0.531	d = −0.30
Fall Time	s	138.8	44.1	14.7	9	153.0	25.3	8.4	9	p = 0.417	d = −0.39
Speed	cm/s	38.3	1.8	0.6	8	40.1	1.7	0.6	8	p = 0.077	d = −0.95

Statistical comparisons between groups were performed using an independent two-sample t-test. n = number of animals sampled.

Open-field motor activity testing was used to assess locomotion, exploratory behavior, and anxiety-related responses in a novel environment (Dataset 2). Rats were placed individually in an enclosed open-field arena and allowed to explore freely for 30 minutes. Activity was recorded using a combination of photobeam sensors and video tracking. Quantified parameters included total distance traveled, average speed, time spent active or resting, number of vertical rears, percentage of time spent in the center versus peripheral zones, and stereotypic movements (Table 4). Testing was conducted three days after the final exposure. No differences were detected between control and MB-exposed animals for any open-field behavioral measure, including average speed, active or resting time, number of rears, time spent in the center zone, or stereotypic movements.

**Table 4:** Motor Behavior Results.

Metric	Unit	Control				MB				p-value	Effect Size
		Mean	SD	SEM	n	Mean	SD	SEM	n		
Distance Traveled	m	349.6	77.3	27.3	8	405.8	71.1	25.2	8	0.153	d = -0.76
Resting Time	s	420.0	207.7	73.4	8	303.3	105.2	37.2	8	0.227	δ = 0.38
Active Time	s	1379.9	207.7	73.4	8	1496.6	105.2	37.2	8	0.227	δ = -0.38
Total Rears	count	169.1	56.9	20.1	8	177.8	42.8	15.1	8	0.733	d = -0.17
Time in Center	%	48.3	3.6	1.2	8	50.6	5.7	2.0	8	0.358	d = -0.47
Fine Movements	count	236.3	29.8	10.5	8	225.2	14.3	5.0	8	0.357	d = 0.48
Total Ambulations	count	6035.6	1512.4	534.7	8	7011.5	1316.2	465.3	8	0.190	d = -0.69

Statistical comparisons between groups were performed using an independent two-sample t-test. n = number of animals sampled.

### Selective atrophy in lumbar motoneurons

**Fig 1:**
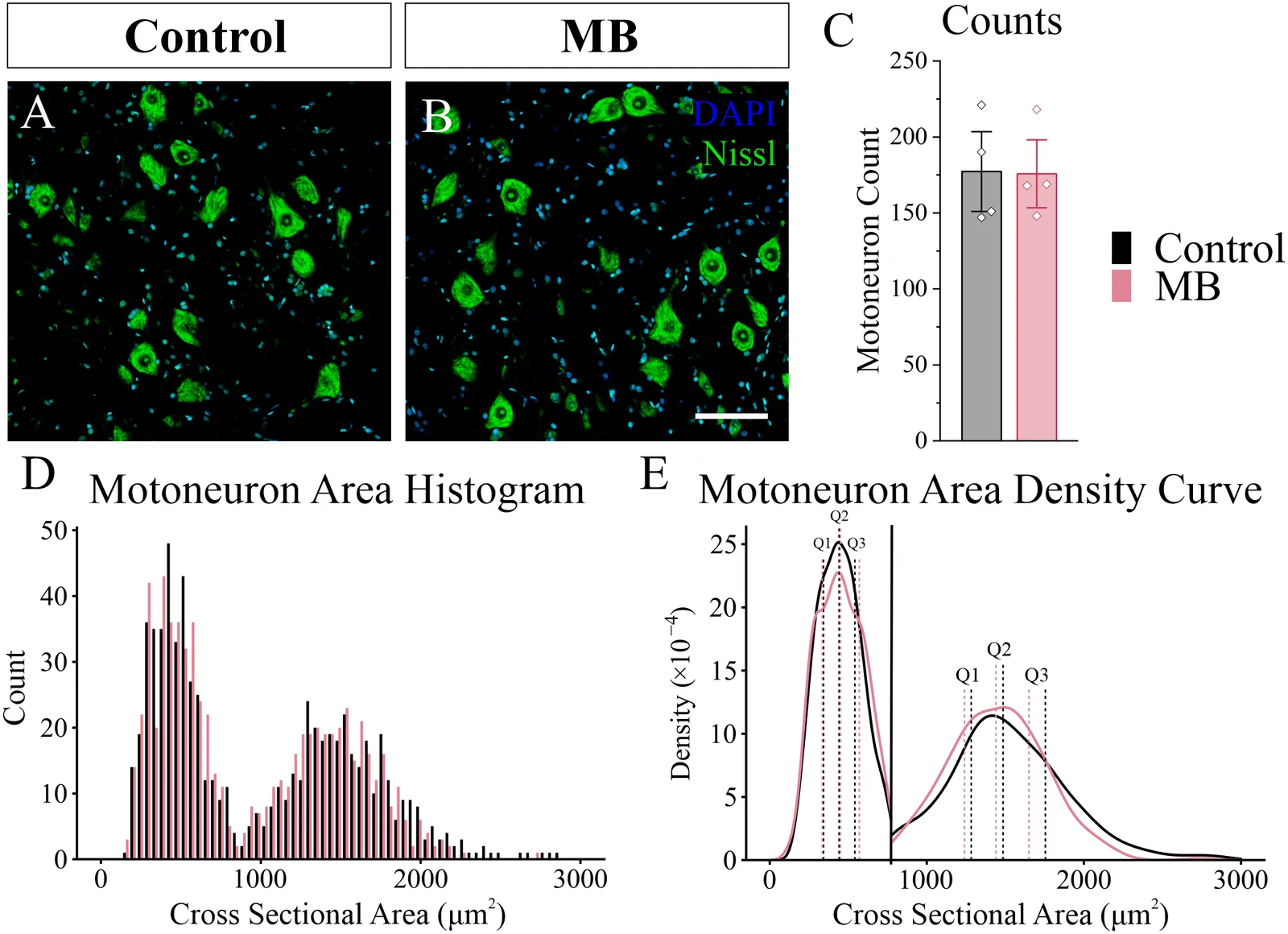
Distribution of spinal motoneuron soma cross-sectional area in control and methyl bromide–exposed animals. (A–B) Representative cross-sectional micrographs of laminae IX to show the motoneuron pool, revealed with NISSL (green) to identify neurons and DAPI (blue) to identify nuclei, in the ventral horn of lower lumbar spinal cord from a control animal (A) and a methyl bromide (MB) exposed animal (B). No obvious qualitative differences were revealed in the micrographs. Scale bar = 100 µm. (C) Motoneuron counts per animal shown as bar graphs (mean ± SD), with diamonds indicating individual animal means. Total motoneuron counts did not differ between groups. (D) Histogram of motoneuron soma cross-sectional area showing the distribution of motoneuron sizes in control (black) and MB exposed (red) animals from 1,416 individual motoneurons measured in 4 control and 4 methyl bromide exposed animals. The distributions demonstrate a bimodal pattern consistent with small and large motoneuron populations. (E) Kernel density estimation of soma cross-sectional area illustrating the bimodal distribution in both groups. Separation of the distribution into small and large modes revealed a leftward shift in the large motoneuron population in methyl bromide–exposed animals relative to controls. Quartiles of each Gaussian mode were compared between groups, along with the rightmost tail of the distribution representing the largest motoneurons.

Motoneuron (MN) degeneration was assessed by quantifying both the number of lumbar MNs and MN soma size as an index of neuronal atrophy (Dataset 3). MNs were identified as large neurons located in lamina IX of the ventral horn within the L4–L6 segments of the lumbar spinal cord (Fig 1A–B). The representative micrographs did not reveal obvious qualitative differences between groups. Therefore, MN number was evaluated using quantitative morphometric analysis, in which MN nuclei were counted in 50-µm sections in 6 unilateral ventral horns, three from the left side and three from the right side and averaged to generate an animal-level estimate. No detectable differences were observed in MN numbers between groups (S2 Table in S1 File) indicating no evidence of MN loss (Fig 1C, Dataset 4).

To assess MN atrophy, soma cross-sectional area was measured for each MN from single optical confocal sections taken through the center of the soma at the level of the nucleolus (Dataset 3). A total of 1,416 lumbar motoneurons (709 control, 707 MB) were analyzed to characterize motoneuron soma size. Because motoneuron morphometry represents a hierarchical dataset in which many neurons are sampled from each animal, soma size was analyzed using linear mixed-effects models that account for clustering of neurons within animals. The distribution of MN soma sizes was bimodal (Fig 1D, S3 Table in S1 File), consistent with previous reports. Across the full MN population, no significant differences in soma cross-sectional area were detected between groups using a log-scale linear mixed-effects model that accounted for neuron-level variability (Fig 1D, S4 Table in S1 File). However, MN properties exist along a continuum, with smaller MNs exhibiting lower activation thresholds and slower, fatigue-resistant firing characteristics, and larger MNs exhibiting higher activation thresholds and faster, more fatigable firing properties. Therefore, to analyze the entire spectrum, we assessed the standard quartiles (Q1, Q2, and Q3) of each mode after modal differentiation of the bimodal distribution (Fig 1E, S5 Table in S1 File, S4 Fig). No significant effects were observed across quartiles in the small area mode, however, evidence of a trend toward decreased cross-sectional area in Q3 of the large mode was present (Control vs. MB difference in Q3 of large-area mode: −95.53 ± 53.36 µm^2^; p = 0.074, quantile mixed-effects model). Finally, based on the evidence previously stated, analysis was restricted to the largest MNs (cross-sectional area > 1500 µm²), and revealed a significant 17.25% reduction in soma area in MB-exposed animals relative to controls (Table 5, p = 0.031, linear mixed-effects model). Collectively, these findings indicate no evidence of MN loss but demonstrate selective atrophy of the largest MNs.

**Table 5:** Soma cross-sectional area of large motoneurons in control and methyl bromide–exposed animals.

Experimental Group	Animals	Neurons	Mean ± SD (µm^2^)	95% CI	Z	Effect Size	p-Value
Control	4	177	1805.68 **±** 281.58				
Methyl Bromide	4	161	1498.43 **±** 274.26	−179.82 to −8.57	−2.16	d = −0.44	0.031

Values represent motoneuron soma cross-sectional areas with the rightmost tail of the distribution representing the largest motoneurons (>1500 µm2) Statistical comparison between groups was performed using a linear mixed-effects model with experimental group as a fixed effect and animal as a random intercept to account for neurons nested within animals. The model was fit on the natural logarithm of soma cross-sectional area using restricted maximum likelihood estimation. P-values represent the fixed-effect test for methyl bromide exposure relative to control.

### No evidence of astrogliosis in the lumbar spinal cord

**Fig 2:**
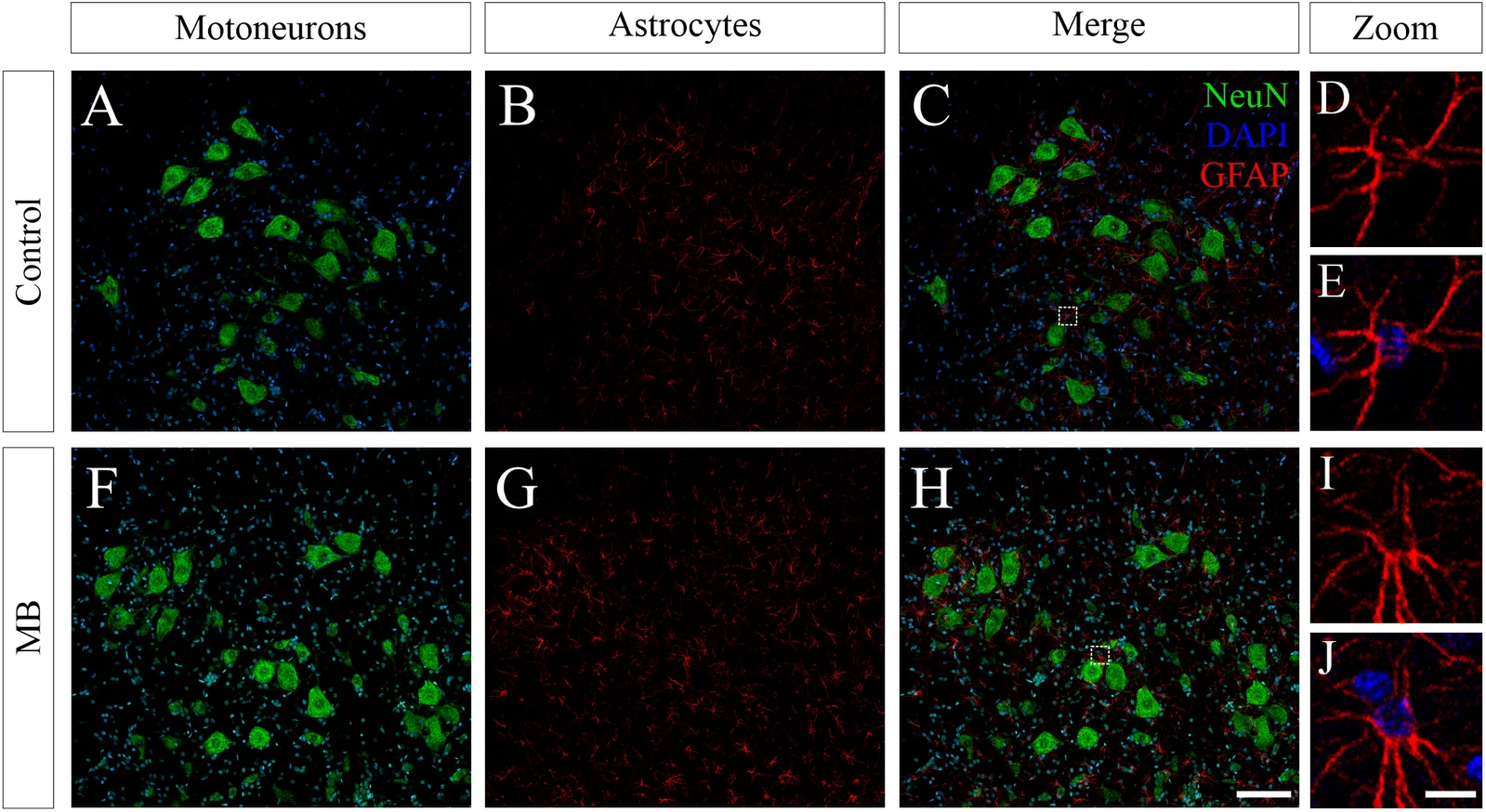
No detectible astrogliosis in lamina IX of the spinal cord following methyl bromide exposure. (A-J) Confocal microscopy images of lamina IX in the lumbar spinal cord from a control animal (A-E) and a MB exposed animal (F-J). Tissue sections are immunolabeled to show neurons (NeuN, green), astrocytes (GFAP, red), and cell nuclei (DAPI, blue). (A, F) Visualization of NeuN-positive motoneurons and other DAPI-stained nuclei. (B, G) Visualization of GFAP-positive astrocytes. (C, H) Merged images showing the spatial relationship between neurons, astrocytes, and other cells. (D, I) Magnified views of GFAP-positive astrocytes from the boxed regions indicated in (C) and (H), respectively. (E, J) Magnified merged-channel views from the same boxed regions, illustrating the astrocytic nuclei visualized by DAPI. Scale bar in (H) = 100 µm (applies to A-C, F-H). Scale bar in (J) = 10 µm (applies to D, E, I, J).

GFAP immunoreactivity was measured in the lumbar spinal cord to assess astrocyte reactivity (Fig 2, Dataset 5). Quantification included mean fluorescence intensity, integrated optical density, and percent area GFAP positive within the defined region of interest (Table 6, Fig 2). No significant differences were detected between rats exposed to 300 ppm MB and control rats for any of the GFAP outcome measures, indicating no evidence of detectable astrogliosis under the conditions examined.

**Table 6:** Comparison of GFAP Immunoreactivity in Lamina IX of the Spinal Cord.

**Assessment**	**Control, n = 4**	**Methyl Bromide, n = 4**	Assumption check / test used	**Difference** **&** **95% CI**	**Effect size**	**p-value**
Integrated Optical Density (lum*µm²)	6,101,017 ± 3,662,841	4,947,178 ± 1,662,050	Raw-scale distributions approximately normal; variance heterogeneity not detected. Welch’s t-test	Mean difference: −1,153,840 (95% CI −6,641,590–4,333,910)	Hedges’ g = −0.35	p = 0.596
Mean Intensity (lum)	23.22 ± 1.28	20.07 ± 3.08	Control group violated normality; log transform did not adequately correct this. Exact Mann–Whitney U	Mean difference: −3.15 (95% CI −7.77 to 1.47)	Rank-biserial r = −0.75	p = 0.114
Percent Area (%)	27.92 ± 6.38	28.97 ± 3.01	Raw-scale distributions approximately normal; variance heterogeneity not detected. Welch’s t-test	Mean difference: 1.05 (95% CI −8.50 to 10.60)	Hedges’ g = 0.18	p = 0.780

Assumptions were evaluated separately for each endpoint by inspection of group distributions and variance structure. When approximate normality and variance compatibility were acceptable, Welch’s two-sample t-test was used and effect size was reported as Hedges’ g. When normality was not adequately supported, the exact Mann–Whitney U test was used and effect size was reported as rank-biserial correlation. Data are presented as mean ± SD, and n = the number of animals sampled.

### Peripheral motor neuropathy in fast-twitch muscle

**Fig 3:**
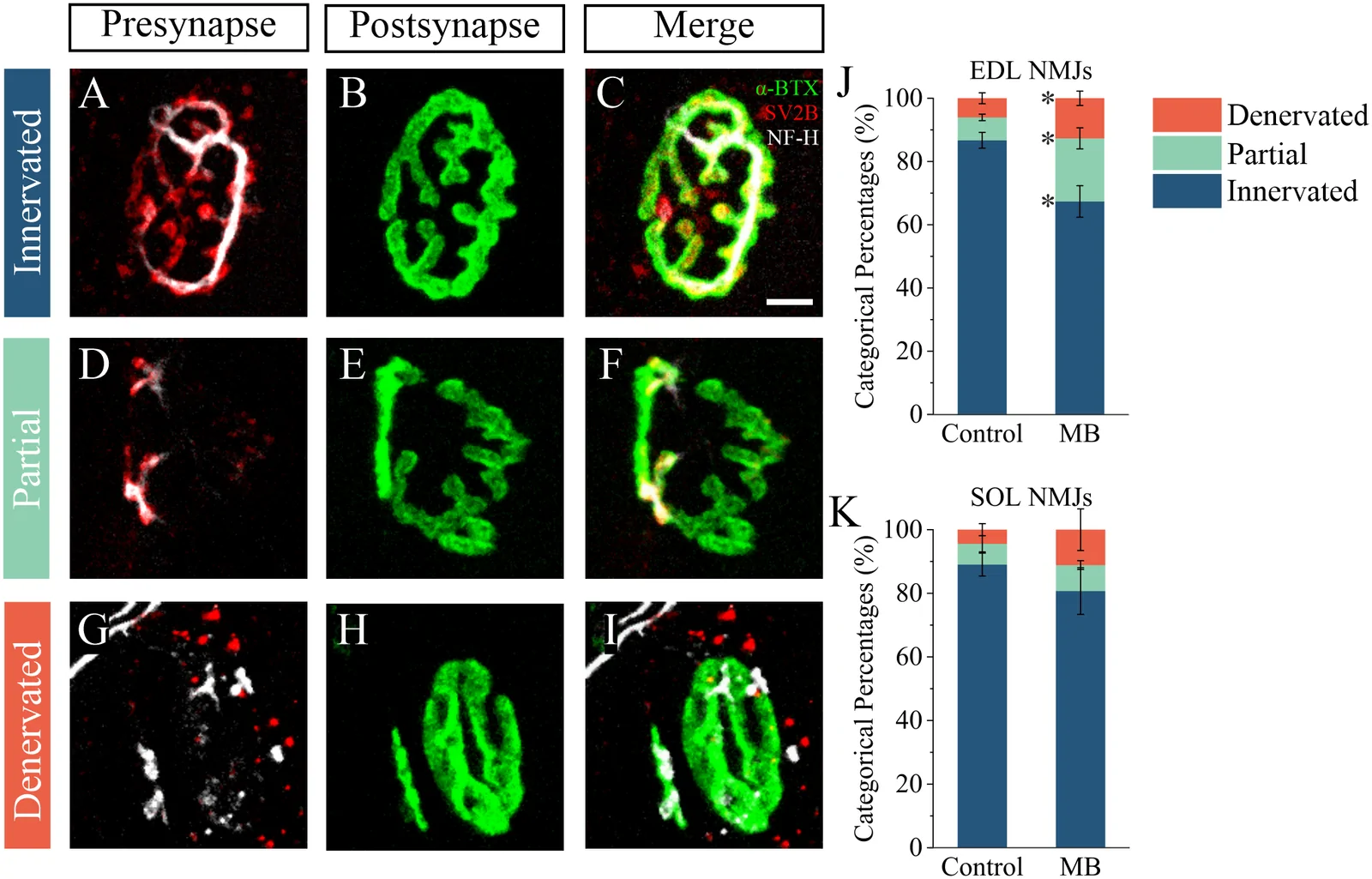
Increased prevalence of denervated motor endplates in fast-twitch muscles following methyl bromide exposure. Neuromuscular junction (NMJ) innervation status in hindlimb muscles was assessed to evaluate structural alterations following methyl bromide (MB) exposure. (A–I) Representative confocal images illustrating NMJ classification categories. (A–C) Innervated NMJs, showing close apposition and structural alignment between presynaptic terminals (A) and postsynaptic end plates (B). (D–F) Partially innervated NMJs, exhibiting reduced overlap between presynaptic (D) and postsynaptic (E) structures. (G–I) Denervated NMJs, characterized by absence of presynaptic–postsynaptic alignment (G–H). (J–K) Quantification of NMJ categories expressed as percentage of total NMJs (mean ± SD). Asterisks indicate significant differences. (J) In extensor digitorum longus (EDL) muscles, the proportion of innervated NMJs decreased, while partially innervated and denervated NMJs increased (p = 0.014, Welch’s t-test; p = 0.016, Welch’s t-test; p = 0.049, Student’s t-test, respectively). A total of 328 EDL NMJs (164 control and 164 MB-exposed) from five animals per group were analyzed. (K) No significant differences in NMJ category distribution were observed in soleus (SOL) muscles. A total of 448 SOL NMJs (242 control from seven animals and 206 MB-exposed from six animals) were analyzed. Scale bar = 20 µm.

Lower lumbar spinal MNs innervate hindlimb skeletal muscles and form synapses with myofibers called neuromuscular junctions (NMJs). Morphological assessment of NMJs provides information on the structural integrity of this synapse, which is critical for normal motor function (Dataset 6). NMJ innervation status was evaluated by assessing the structural organization of presynaptic and postsynaptic components. Presynaptic terminals were labeled with antibodies against neurofilament heavy chain (NF-H), a structural axonal protein, and synaptic vesicle protein 2B (SV2B), a vesicular protein associated with neurotransmitter-containing synaptic vesicles. Postsynaptic structures were labeled with fluorescently conjugated α-bungarotoxin (αBTX), which binds to nicotinic acetylcholine receptors on the muscle endplate (Fig 3A–3I). In fully innervated NMJs, presynaptic markers (NF-H and SV2B) closely overlap with postsynaptic αBTX labeling and exhibit similar morphological organization (Fig 3A–3C). Disruption of this alignment results in distinct structural phenotypes that can be classified as partially denervated, characterized by reduced overlap between presynaptic and postsynaptic structures (Fig 3D–3F), or fully denervated, characterized by the absence of presynaptic signal at the postsynaptic endplate (Fig 3G–3I).

Innervation status was quantified in the extensor digitorum longus (EDL), a predominantly fast-twitch muscle, and the soleus (SOL), a predominantly slow-twitch muscle (Table 7, Dataset 7). Quantitative analysis included a total of 328 EDL NMJs (164 control and 164 MB-exposed) and 448 SOL NMJs (242 control and 206 MB-exposed) analyzed. The EDL dataset was obtained from five animals per group, whereas the SOL dataset was obtained from seven control animals and six MB-exposed animals. In the EDL, the proportion of fully innervated NMJs was significantly reduced in MB–exposed animals compared with controls. Correspondingly, the proportions of partially denervated and fully denervated NMJs were significantly increased. In contrast, no significant differences in NMJ innervation status were observed in the SOL muscle.

**Table 7:** Percent Motor Innervation and Denervation of the Neuromuscular Junction in Select Hindlimb Skeletal Muscles.

Metric	Muscle	Control				MB				p-value	Effect Size
		Mean	±SD	SEM	n	Mean	±SD	SEM	n		
Innervated Percentage	Extensor Digitorum Longus	86.71%	5.59%	2.50%	5	67.37%	11.16%	4.99%	5	p = 0.014	d = 2.19
	Soleus	89.03%	9.50%	3.59%	7	80.71%	17.96%	7.33%	6	p = 0.341	d = 0.59
Partial Percentage	Extensor Digitorum Longus	7.25%	2.31%	1.03%	5	19.96%	7.46%	3.34%	5	p = 0.016	d = −2.30
	Soleus	6.86%	7.39%	2.79%	7	8.15%	3.39%	1.38%	6	p = 0.691	d = −0.22
Denervated Percentage	Extensor Digitorum Longus	6.04%	3.89%	1.74%	5	12.67%	5.05%	2.26%	5	p = 0.049^**†**^	d = −1.47
	Soleus	4.43%	5.02%	1.90%	7	11.14%	16.03%	6.55%	6	p = 0.364	d = −0.58

### Altered synaptic structure of neuromuscular junctions

**Fig 4:**
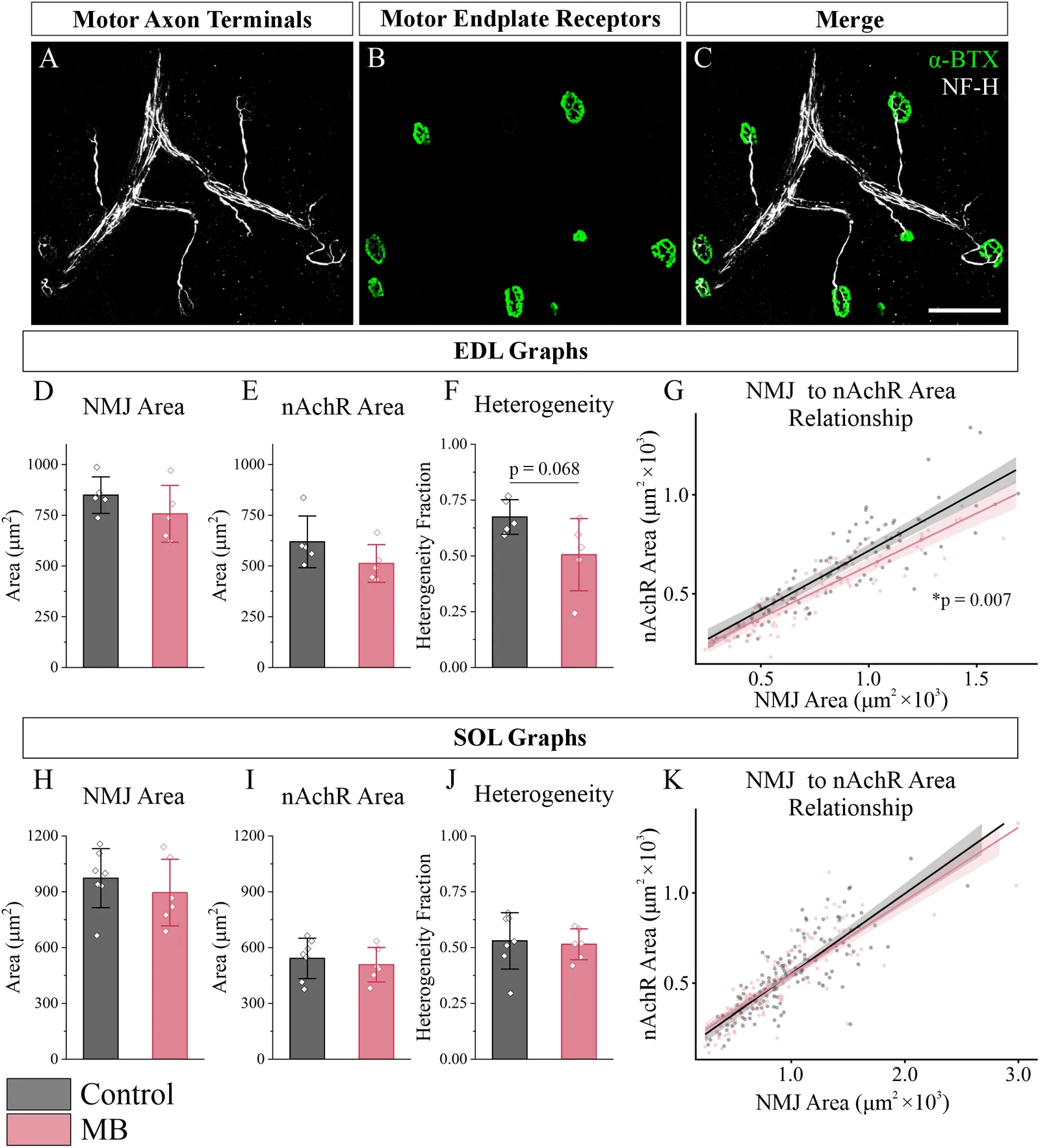
Altered postsynaptic nicotinic acetylcholine receptor morphology in fast-twitch muscle following methyl bromide exposure. Postsynaptic nicotinic acetylcholine receptor (nAChR) organization at neuromuscular junctions (NMJs) was assessed following methyl bromide (MB) exposure. (A–C) Representative confocal images of an efferent motor axon branch innervating multiple myofibers. Presynaptic structures were labeled by neurofilament heavy chain (NF-H) immunoreactivity (A), and postsynaptic nAChRs were labeled with fluorescent α-bungarotoxin (αBTX) (B); merged image shown in (C). Scale bar = 100 µm. (D–G) Quantitative analysis of NMJ morphology in extensor digitorum longus (EDL) muscles. No differences were observed in mean NMJ area (D) or mean nAChR area (E). (F) Postsynaptic pixel heterogeneity showed a non-significant decrease (p = 0.068). (G) Relationship between nAChR area and NMJ area; nAChR area at the mean NMJ area was reduced in MB-exposed EDL muscles (p = 0.007), with no difference in slopes between groups. (H–K) Corresponding analyses in soleus (SOL) muscles. No differences were observed in mean NMJ area (H), mean nAChR area (I), postsynaptic pixel heterogeneity (J), or the relationship between nAChR area and NMJ area (K). Overall, A total of 200 EDL NMJs (100 control and 100 MB-exposed) from five animals per group and 260 SOL NMJs (140 control from seven animals and 120 MB-exposed from six animals) were analyzed.

Consistent with previously described quantitative morphometric approaches for NMJ analysis [33–36], we measured NMJ area, absolute nAChR area, and nAChR pixel heterogeneity to assess structural changes induced by MB exposure (Fig 4, Dataset 6). The post synaptic nAchR structural organization is inherently heterogeneous, with each skeletal muscle fiber containing a single endplate that represents the synaptic contact between an individual motoneuron and its corresponding muscle fiber (Fig 4A–C).

Initial comparisons using two-sample t-tests detected no significant differences in NMJ area, absolute nAChR area, or nAChR pixel heterogeneity in the EDL muscle (Fig 4D–F, S6 Table in S1 File). Furthermore, nAchR fragmentation was unaltered by MB exposure (S5 Fig, S9 Table in S1 File). However, because postsynaptic nAChR area is strongly related to overall NMJ size, we further evaluated this relationship using a linear mixed-effects model that accounted for NMJ area while accommodating the hierarchical structure of the dataset. This analysis demonstrated that nAChR area was significantly reduced in MB-exposed EDL muscles for NMJs of equivalent size (MB effect on EDL nAChR area = −66.93 ± 22.75 µm², *p* = 0.006; Fig 4H, Tables S7–S8 in S1 File). The model also identified a significant positive relationship between nAChR area and NMJ area (EDL nAChR–NMJ scaling slope = 0.597 ± 0.036 µm²/µm², *p* = 2.857 × 10^-^⁴⁹), with no evidence that MB altered this scaling relationship (MB × NMJ area interaction = −0.069 ± 0.050 µm²/µm², *p* = 0.171). Collectively, these findings indicate that MB exposure produced an approximately 70 µm² reduction in postsynaptic nAChR area across the full range of EDL NMJ sizes, without altering the normal relationship between nAChR area and NMJ size. In contrast, no significant differences, except for nAchR fragment count, were detected in the SOL muscle using either two-sample *t*-tests or linear-mixed effects modeling of the relationship between nAchR area and NMJ area (Fig 4H–K, Tables S6–S8 in S1 File). The nAchR fragment count was significantly reduced in MB exposed SOL muscles (mean fragment counts = Control: 1.720 ± 0.06 vs. MB: 1.367 ± 0.06, *p* = 5.859 × 10^−5^, Wald z-test). No other significant differences were detected across post synaptic morphology metrics in the SOL (S5 Fig, S9 Table in S1 File).

We also measured the nerve terminal regions of each NMJ, visualized by the combination of SV2B and NFH immunoreactivity (S5 Fig, S9 Table in S1 File, Dataset 6). These data were assessed entirely using mixed effect modeling, and changes are reported as either fold-change (FC) on the natural log-scale, or odds-ratio (OR). Nerve terminal area was significantly decreased in EDL muscle exposed to MB (FC = 1.529, 95% CI: [1.168, 2.001], p = 0.002, t-test), while remaining conserved in SOL muscle (FC = 0.888, 95% CI: [0.679, 1.161], p = 0.545, t-test). The probability of presynaptic+ NMJs was also quantified, using a binomial framework. This revealed a significantly reduced rate of presynaptic+ NMJs in EDLs exposed to MB (OR = 10.086, 95% CI: [2.285, 44.524], p = 0.005, Wald z-test). Again, no change was observed in the SOL under the same analysis (OR = 1.537, 95% CI: [0.382, 6.178], p = 0.374, Wald z-test).

## Discussion

Methyl bromide (MB) remains a relevant environmental and occupational toxicant used worldwide for pest control in commodity storage, shipping applications, and quarantine protocols, where accidental or occupational exposures may be unpredictable and difficult to monitor. Although the acute toxicity of MB is well recognized, the neurological consequences of repeated, low-level exposure remain poorly defined, highlighting the need for studies capable of detecting subtle or early indicators of neurotoxicity. In the present study, adult rats were exposed to either clean air or 300 ppm MB by nose-only inhalation for 2 hours/day, 5 days/week, for 4 weeks. Exposed rats showed no clinical signs of intoxication, no deficits in motor performance, and no evidence of spinal MN degeneration or spinal astrogliosis. Despite the absence of measurable functional impairment or overt central pathology, we identified subtle peripheral neuromuscular abnormalities at the NMJ, including reduced end-plate occupancy. These findings support the conclusion that peripheral motor terminals represent a sensitive target for MB toxicity and that subclinical injury can occur in the absence of overt symptomatology. Collectively, these results indicate that extended exposure may drive progression of these early neuromuscular changes toward overt dysfunction, although the potential for recovery following exposure cessation remains to be determined.

Multiple experimental and clinical reports have identified the peripheral nervous system as a sensitive target for MB toxicity. Previous rodent studies have shown dose-dependent impairment of peripheral nerve conduction, axonal swelling, and Schwann cell degeneration following repeated inhalation exposures at concentrations of 200–300 ppm [12,20,37]. Histopathological evaluation has further demonstrated axonopathy accompanied by demyelination and glial activation, even in the absence of overt behavioral impairment [12,38–41]. Human case reports similarly describe distal sensorimotor neuropathies, paresthesia, and reduced nerve conduction velocity in workers with documented or suspected overexposure, supporting concordance between animal and human findings [2,16,17,38]. Collectively, these data indicate that peripheral nerves are a sensitive site of MB-induced injury and that subclinical neuropathy may precede clear clinical manifestations or measurable functional deficits.

At the NMJ, MB-induced effects were not uniformly distributed across muscle types. MB-induced structural alterations were more pronounced in EDL than in soleus NMJs, suggesting that MB may preferentially affects specific motor unit populations rather than individual tissues in isolation. This pattern is further supported by the concurrent atrophy observed in the largest population of spinal MNs, which is consistent with preferential involvement of MNs that innervate fast-twitch motor units. However, because only two muscles representing distinct fiber-type compositions were examined, the present study cannot determine whether this pattern reflects an inherent vulnerability of specific motor unit types or the particular exposure conditions used.

Measures of innervation quality and the probability of presynaptic presence were reduced in the EDL, whereas alterations in the soleus were modest. In addition, the EDL muscle exhibited reduced nAchR area when comparing across equivalent NMJ areas, as well as reduced nerve terminal area. A reduction in nAchR area may be consistent with denervation-associated postsynaptic receptor remodeling [42], although reduced nAchR area could also reflect changes in muscle fiber caliber. Given that NMJ morphology is influenced by both fiber size and fiber type, the present data cannot distinguish whether this reflects synaptic loss, myofiber atrophy, or compensatory remodeling. Although a shift in muscle fiber phenotype could contribute to the observed differences, muscle fiber typing was not performed and this possibility remains speculative.

One possible mechanism contributing to the preferential involvement of the EDL is oxidative stress, which has been implicated in both MB-induced neurotoxicity and skeletal muscle atrophy [43–45]. However, the relationship between oxidative stress and fiber-type vulnerability is complex. Some studies suggest that slow-twitch muscle may be preferentially affected under certain oxidative stress conditions [46], whereas others indicate that greater reactive oxygen species production in fast-twitch fibers may increase their susceptibility to injury [47]. Consequently, the present findings do not establish oxidative stress as the mechanism underlying the observed muscle-specific differences but instead suggest that differential motor unit susceptibility warrants further investigation. This concept may have particular relevance to aging, which is characterized by progressive motor unit remodeling, denervation, increased oxidative stress, impaired reinnervation, and preferential vulnerability of fast motor units. Thus, older animals may exhibit greater sensitivity to MB-induced neuromuscular injury than the young adults examined in the present study. Evaluation of age-dependent susceptibility represents an important direction for future investigation.

The neuromuscular symptoms reported in human MB intoxication, including weakness, ataxia, and myoclonus, are consistent with dysfunction somewhere along the motor pathway [4,48]. However, the present findings do not establish whether NMJ remodeling represents a primary site of MB toxicity or develops secondary to more proximal peripheral nerve injury. Likewise, the disproportionate effect on the EDL should not be interpreted as evidence that MB selectively targets fast-twitch motor units. Rather, it demonstrates that structural abnormalities were more readily detected in this muscle under the exposure conditions examined.

Evidence specifically linking MB toxicity to structural disruption of the NMJ remains limited. Previous animal studies have described peripheral axonal injury and distal degeneration following MB exposure, but they generally did not assess NMJ integrity using contemporary structural approaches such as fluorescent synaptic labeling and end-plate morphometry. Human case reports of severe intoxication have documented flaccid weakness and areflexia, findings compatible with lower MN or peripheral motor dysfunction, but they do not provide direct morphological evidence of NMJ denervation [17,38,41,49,50]. In this context, the present findings extend the existing literature by identifying structural abnormalities at the NMJ and suggest that synaptic pathology may represent an under-recognized component of MB-induced peripheral neurotoxicity. Future studies incorporating additional muscle groups, muscle fiber typing, electrophysiological assessments, longitudinal time points, and multiple exposure levels will be important for determining whether differential motor unit susceptibility represents a reproducible feature of MB neurotoxicity and for defining the mechanisms underlying these changes.

Prior toxicology studies in rodents have reported variable responses to MB, reflecting differences in species, exposure concentration, route, daily exposure duration, cumulative exposure, and study design. Consequently, exposure concentration alone is insufficient to compare toxicity across exposures. For example, repeated inhalation exposures of approximately 200–300 ppm for 4 hours a day have been associated with peripheral nerve dysfunction, altered motor behavior, and abnormal sensorimotor responses [19,32]. However, these effects were not uniformly observed across all behavioral endpoints or animals. In one study, exposure to 300 ppm MB produced impaired rotarod performance and transient reduced spontaneous motor activity, yet open-field measures such as rearing were unaffected and histologic examination failed to demonstrate corresponding lesions in the nervous system [19]. Other studies have produced minimal or no measurable effects following lower concentration chronic inhalation regimens despite prolonged exposure (55 ppm for up to 36 weeks) [51]. Targeted nasal deposition inhalation studies have also shown marked dose-dependent injury in the olfactory epithelium, underscoring the importance of local tissue susceptibility [52–54].

The present study used repeated nose-only inhalation of 300 ppm MB for 2 hours/day, 5 days/week, for 4 weeks, a regimen intended to model repeated subacute, sublethal inhalation exposure. Although the exposure concentration was comparable to previous studies, the daily exposure duration was half that used in the repeated 300-ppm inhalation studies of Ikeda et al. and Kato et al. [19,32]. Although cumulative chamber exposure is not equivalent to internal dose because MB uptake, distribution, metabolism, and clearance are influenced by toxicokinetics and exposure conditions, this comparison illustrates that identical airborne concentrations do not necessarily represent equivalent toxicological exposures. The shorter daily exposure duration may also have permitted greater toxicant clearance and recovery between exposure sessions. Furthermore, although the NOES provided a continuously replenished inhalation atmosphere under controlled flow and pressure conditions, respiratory ventilation and internal biomarkers were not measured during exposure. Consequently, the relationship between chamber concentration, inhaled dose, and internal dose could not be directly quantified and should be considered a limitation of the present study.

Under these conditions, animals did not exhibit overt clinical toxicity, weight loss, or detectable behavioral impairment. Nevertheless, mild peripheral neuromuscular abnormalities were detected using targeted assessments. These findings are consistent with the hypothesis that the present exposure regimen produced subtle peripheral neurotoxic effects that remained below the threshold required to impair integrated motor behavior. Whole-body behavioral assays generally reflect the coordinated function of multiple neural systems and may therefore be less sensitive than targeted morphological analyses for detecting localized structural abnormalities. Rather than contradict previous reports of MB neurotoxicity, the present findings extend the exposure-response relationship by demonstration that repeated subacute inhalation exposure can produce measurable peripheral neuromuscular changes even when conventional clinical or behavioral endpoints remain normal [28,40,53,54].

Mechanistically, MB is a highly reactive alkylating agent that can modify nucleophilic targets in proteins, lipids, and nucleic acids. Its toxicity has been linked to glutathione-dependent detoxification pathways, and repeated exposure may compromise antioxidant defenses by depleting glutathione reserves [10]. Experimental work further suggests that MB can disrupt mitochondrial function and promote oxidative stress, processes that may impair axonal metabolism and contribute to distal neural injury [10,15,27,40,55–58]. Peripheral axons and associated Schwann cells may be especially vulnerable because of their high metabolic demands and dependence on long-distance axonal transport [2,38,39]. Glial activation and limited demyelinating changes have also been reported in some exposure models, indicating that MB toxicity may involve both neuronal and non-neuronal components [40,53,54]. MB toxicity has also been proposed to cause cholinesterase inhibition via methyl phosphate synthesis, but this explanation remains speculative and less established than aberrant alkylation of alternative targets leading to systemic oxidative stress [9]. If present, cholinesterase inhibition could increase cholinergic signaling and thereby contribute to motor dysfunction, particularly at higher levels of exposure. Some signs reported following repeated low-concentration MB exposure, including salivation, tremor, nausea, and vomiting, are also consistent with cholinergic excess [14]. However, these manifestations are not specific to cholinesterase inhibition, and the present findings do not directly support this mechanism. Accordingly, cholinergic dysfunction should be considered a possible, but not primary, explanation for the motor-related effects observed after repeated low-level MB exposure.

Several limitations should be considered when interpreting the present findings. First, respiratory ventilation and internal biomarkers of MB exposure were not measured during the present study, precluding direct quantification of inhaled or systemic dose. Nevertheless, previous validation studies performed using the same NOES platform demonstrated sustained minute ventilation in acclimated rats during prolonged restraint, suggesting that marked reductions in ventilation due to restraint are unlikely following acclimation. Because these measurements were obtained using a different restraint configuration and were not collected as part of the present study, they were not used to estimate inhaled dose. Second, because only a single exposure regimen and terminal time point were evaluated, the temporal relationship between peripheral neuromuscular abnormalities and behavioral function cannot be established. Consequently, the present findings should not be interpreted as demonstrating that NMJ alterations necessarily precede behavioral impairment. Rather, they indicate that mild peripheral neuromuscular abnormalities were detectable under exposure conditions in which overt behavioral deficits were not observed. Finally, although quantitative histological analyses identified significant structural changes, additional studies incorporating multiple exposure levels, longitudinal assessments, electrophysiological measurements, respiratory physiology, and internal dosimetry will be important to define the exposure-response relationship and determine the threshold at which peripheral abnormalities progress to measurable functional impairment.

Overall, these results indicate that repeated MB exposure can produce mild peripheral neuromuscular toxicity in the absence of overt clinical signs or neurobehavioral deficits. The apparent selective vulnerability of distal motor terminals and synaptic structures, together with the absence of more overt functional impairment, suggests that the NMJ may be a sensitive target of MB neurotoxicity under the exposure conditions employed. From an occupational health perspective, these findings raise the possibility that exposed workers could sustain subclinical peripheral neurotoxic injury despite preserved performance and limited symptom reporting. This reinforces the need for exposure reduction and mitigation strategies, as well as surveillance or monitoring approaches that do not rely exclusively on clinical symptoms. Future studies incorporating electrophysiology, muscle fiber typing, longitudinal peripheral biomarkers, and internal dosimetry will be important for determining whether these subtle neuromuscular changes resolve, progress, or accumulate during chronic exposure and for defining the relationship between exposure, internal dose, and functional outcome.

## Supporting information

S1 File Supplemental Tables. (DOCX)

S1 Fig Body weight trajectories did not differ between experimental groups. Weekly body weights were measured in control (gray) and methyl bromide–exposed (MB, red) rats throughout the study. Inhalation exposures occurred during weeks 3–7, and weeks 7–8 represent the post-exposure period. Animals were enrolled in two staggered cohorts, with four control and four MB-exposed rats beginning the study one week after the initial cohort of five control and five MB exposed rats. No significant differences in body weight were detected between groups at any time point (Kruskal–Wallis test; group means and p values are reported in Supplemental Table 1). Data are presented as mean ± SEM; n = 9 rats per group, except n = 4 per group at week 8.
(TIF)

S2 Fig Kinetic weight bearing limb lateralization of front paws. Forest plot of front paw lateralization results from gait analysis. Front limb lateralization was quantified for each animal across all metrics as left limb value minus right limb value. Lateralization values were then analyzed in R using a linear mixed effect model (R packages: *lme4, lmerTest*) with experimental group, limb, and their interaction as fixed effects, with animal identification accounted for as a random intercept. Group contrasts were derived from the model as estimated marginal means (R package: *emmeans*). Effect sizes were calculated by dividing the estimated marginal mean of each contrast by the residual standard deviation from the fitted model (Cohen’s d style effect size, reapplied to model fitted values). Control group is shown in black, and MB in red. A negative effect size indicates the right paw value was larger on average, and a positive effect size represents the left paw value was larger. The 95% confidence intervals were derived from model-estimated marginal means and Satterthwaite-adjusted degrees of freedom. P-values reflect whether estimated marginal means differ from zero, using model-based t-tests.
(TIF)

S3 Fig Kinetic weight bearing limb lateralization of rear paws. Forest plot of rear paw lateralization results from gait analysis. Rear limb lateralization was quantified for each animal across all metrics as left limb value minus right limb value. Lateralization values were then analyzed in R using a linear mixed effect model (R packages: *lme4, lmerTest*) with experimental group, limb, and their interaction as fixed effects, with animal identification accounted for as a random intercept. Group contrasts were derived from the model as estimated marginal means (R package: *emmeans*). Effect sizes were calculated by dividing the estimated marginal mean of each contrast by the residual standard deviation from the fitted model (Cohen’s d style effect size, reapplied to model fitted values). Control group is shown in black, and MB in red. A negative effect size indicates the right paw value was larger on average, and a positive effect size represents the left paw value was larger. The 95% confidence intervals were derived from model-estimated marginal means and Satterthwaite-adjusted degrees of freedom. P-values reflect whether estimated marginal means differ from zero, using model-based t-tests.
(TIF)

S4 Fig Modal differentiation using gaussian mixture modeling. Representative graphs that demonstrate gaussian mixture modeling and modal differentiation methods. Distributions that were suspected to have a latent structure were statistically evaluated for multimodality using Hartigan’s dip test and Gaussian mixture modelling. For each dataset, Gaussian mixture models (G = 1–3 components) were fit using maximum likelihood, and the best fit of the three was determined using the Bayesian Information Criterion (BIC), with the lowest BIC indicating the best fit. For datasets where two component modeling was most favorable, component mean, variances, and mixing proportions were estimated. A cutoff value was defined as the intersection between the fitted Gaussian components.
(TIF)

S5 Fig Neuromuscular Junction Presynaptic Morphology and Post Synaptic Fragmentation. (A-L) Representative confocal images of NMJs from the Extensor Digitorum Longus (EDL) and Soleus (SOL) muscles of control and methyl bromide (MB) exposed animals. Postsynaptic acetylcholine receptors (nAChRs) are labeled with α-bungarotoxin (aBTX, green), while presynaptic nerve terminals and their axons are identified by immunoreactivity to synaptic vesicle protein 2B (SV2B, red) and neurofilament heavy chain (NFH, white), respectively. Scale bars = 10 µm. (M, P) Quantification of synaptic areas for EDL (M) and SOL (P) muscles. Stacked bar graphs show the mean (± 95% CI) for the total NMJ area (full bar height), with the constituent nAChR area (green) and nerve terminal area (red) displayed within. Individual data points for each animal are shown as correspondingly colored circles. MB exposed EDLs show reduced nAChR area (p = 0.012, t-test), and nerve terminal area (p = 0.002, t-test). (N, Q) Histograms showing the distribution of nAChR fragmentation, represented by the number of distinct nAChR regions per NMJ, for EDL (N) and SOL (Q) muscles in control (black) and MB-exposed (red) groups. (O, R) Frequency distribution (bar graphs, left y-axis) and corresponding cumulative percentage (step-wise line plots, right y-axis) of presynaptic nerve terminal areas for EDL (O) and SOL (R) muscles. The plots compare control (black) and MB-exposed (red) groups, illustrating the proportion of fully denervated NMJs (presynaptic area of 0 µm²). The EDL showed a significantly decreased proportion of presynaptic+ NMJs, indicated by an elevation of 0 values along the presynaptic area axis (p = 0.005, Wald z-test). A total of 460 NMJs were analyzed for all metrics, except Nerve Terminal Area (M, P), which only analyzed the 403 presynaptic+ NMJs.
(TIF)
