# Supplementary material for: Neuromuscular effects of repeated exposure to the pesticide methyl bromide
Source: PLoS One. 2026 Aug 31;21(8):e0357385. doi: 10.1371/journal.pone.0357385 (PMC13528985; doi:10.1371/journal.pone.0357385)
Supplement: Supplementary file 1 [file pone.0357385.s001.docx]

Methyl Bromide Manuscript – Supplemental Information

Table of Contents: Supplemental Tables

[**Table S1. Mean Body Weights by Date**	2](#_Toc236406063)

[**Table S2. Motoneuron Morphology Results with Two-Sample t-test analysis**	2](#_Toc236406064)

[**Table S3. Bimodality Results for Motoneuron Cross-Sectional Area**	3](#_Toc236406065)

[**Table S4. Motoneuron Cross Sectional Area Mixed-Effects Model Results**	3](#_Toc236406066)

[**Table S5. Quantile Mixed-Effects Model Results for Motoneuron Cross-Sectional Area**	4](#_Toc236406067)

[**Table S6. Neuromuscular Junction Postsynaptic Receptor Morphology Results**	4](#_Toc236406068)

[**Table S7. Linear Mixed Effects Model of Receptor Area as a function of NMJ Area**	5](#_Toc236406069)

[**Table S8. Model-Estimated Means from the Receptor-NMJ Area Mixed-Effects Model**	5](#_Toc236406070)

[**Table S9. Mixed Model Summary of Neuromuscular Junction Morphology Metrics**	6](#_Toc236406071)

# Supplemental Tables

**Table S1. Mean Body Weights by Date**

| **Date Weighed** | **Control** | | | |  | **MB** | | | | **p-value** | **Effect Size** |
| --- | --- | --- | --- | --- | --- | --- | --- | --- | --- | --- | --- |
|  | Mean | SD | SEM | n |  | Mean | SD | SEM | n |  |  |
| 2022-08-02 | 103.85 | 5.62 | 1.87 | 9 |  | 103.98 | 6.66 | 2.22 | 9 | p = 0.965 | d = -0.02 |
| 2022-08-12 | 206.45 | 8.55 | 2.85 | 9 |  | 207.61 | 18.69 | 6.23 | 9 | p = 0.868 | d = -0.08 |
| 2022-08-15 | 236.36 | 11.50 | 3.83 | 9 |  | 236.88 | 22.29 | 7.43 | 9 | p = 0.951 | d = -0.03 |
| 2022-08-22 | 288.08 | 18.41 | 6.14 | 9 |  | 292.16 | 27.29 | 9.10 | 9 | p = 0.715 | d = -0.18 |
| 2022-08-29 | 331.50 | 25.02 | 8.34 | 9 |  | 335.89 | 30.02 | 10.01 | 9 | p = 0.740 | d = -0.16 |
| 2022-09-06 | 381.61 | 36.49 | 12.16 | 9 |  | 380.02 | 34.20 | 11.40 | 9 | p = 0.925 | d = 0.05 |
| 2022-09-12 | 408.97 | 40.69 | 13.56 | 9 |  | 407.11 | 36.08 | 12.03 | 9 | p = 0.919 | d = 0.05 |
| 2022-09-19 | 442.99 | 38.87 | 19.44 | 4 |  | 460.32 | 33.25 | 16.63 | 4 | p = 0.523 | d = -0.48 |

Statistical comparisons between groups were performed using an independent two-sample t-test. Effect size reported as Cohen’s d. Please note that animals were staggered started with exposures, with half the animals beginning a week before the remaining animals. Likewise, endpoint assessments were staggered a week as well.

**Table S2. Motoneuron Morphology Results with Two-Sample t-test analysis**

| **Metric** | **Unit** | **Size** | **Control** | | | |  | **MB** | | | | **p-value** | **Effect Size** |
| --- | --- | --- | --- | --- | --- | --- | --- | --- | --- | --- | --- | --- | --- |
|  |  |  | Mean | SD | SEM | n |  | Mean | SD | SEM | n |  |  |
| Cross Sectional Area | µm^2^ | Large | 1509.89 | 53.38 | 26.69 | 4 |  | 1459.54 | 67.64 | 33.82 | 4 | p = 0.336 | d = 0.75 |
|  |  | Small | 451.12 | 24.06 | 12.03 | 4 |  | 456.81 | 40.83 | 20.41 | 4 | p = 0.820 | d = 0.17 |
| Perimeter | µm | Large | 156.63 | 3.62 | 1.81 | 4 |  | 154.85 | 4.26 | 2.13 | 4 | p = 0.548 | d = 0.45 |
|  |  | Small | 84.89 | 2.26 | 1.13 | 4 |  | 85.7 | 5.24 | 2.62 | 4 | p = 0.790 | d = 0.20 |
| Diameter | µm | Large | 48.08 | 1.17 | 0.58 | 4 |  | 47.28 | 1.2 | 0.6 | 4 | p = 0.375 | d = 0.68 |
|  |  | Small | 26.02 | 0.63 | 0.31 | 4 |  | 26.18 | 1.58 | 0.79 | 4 | p = 0.862 | d = 0.13 |
| Count | count | Total | 29.54 | 5.84 | 2.92 | 4 |  | 29.46 | 4.92 | 2.46 | 4 | p = 0.950 | d = 0.05 |

Statistical comparisons between groups were performed using an independent two-sample t-test. Effect size reported as Cohen’s d.

**Table S3. Bimodality Results for Motoneuron Cross-Sectional Area**

| **Expt. Group** | **Animal ID** | **Shapiro-Wilk p-value** | **Hartigan’s Dip p-value** | **Estimated Gaussian Components** | **Intersection points between Components (µm^2^)** |
| --- | --- | --- | --- | --- | --- |
| Control | 15 | p < 0.001 | p < 0.001 | 2 | 712.14 |
| Control | 17 | p < 0.001 | p = 0.001 | 2 | 784.95 |
| Control | 23 | p < 0.001 | p = 0.001 | 2 | 831.94 |
| Control | 21 | p < 0.001 | p = 0.017 | 2 | 760.37 |
| MB | 42 | p < 0.001 | p < 0.001 | 2 | 851.60 |
| MB | 44 | p < 0.001 | p = 0.035 | 2 | 826.40 |
| MB | 26 | p < 0.001 | p < 0.001 | 3 | 806.62 |
| MB | 46 | p < 0.001 | p < 0.001 | 2 | 758.81 |

Results from multimodality assessment of MN cross-sectional area distributions from all animals. Shapiro-Wilk test indicated all samples were not normally distributed, Hartigan’s Dip test indicated all samples showed evidence of multimodality, and Gaussian mixture modeling predicted a two components mixture was the best fit for all distributions except animal ID 26.

**Table S4. Motoneuron Cross Sectional Area Mixed-Effects Model Results**

| **Group** | **Animals** | **Neurons** | **Mean ± SD (µm^2^)** | **Median Area (µm^2^)** | **p--value** |
| --- | --- | --- | --- | --- | --- |
| Control | 4 | 709 | 991.2 ± 605.4 | 799.6 | p = 0.726 |
| MB | 4 | 707 | 950.8 ± 551.3 | 764.0 |  |

Motoneuron soma cross-sectional area was analyzed using a linear mixed-effects model with exposure group as a fixed effect and animal as a random intercept. In the primary analysis of log-transformed area, Methyl Bromide exposure was not associated with a significant difference in soma size relative to Control ($\beta=-0.0219$, SE = 0.0625, z = -0.350, p = 0.726), corresponding to an estimated 2.16% decrease in soma area (95% CI, 13.44% decrease to 10.59% increase). The sensitivity analysis on the raw scale was concordant ($\beta=-33.91$ µm², SE = 50.82, z = -0.667, p = 0.505).The standardized effect size for the difference in soma cross-sectional area between Methyl Bromide and Control groups was negligible (Cohen’s d ≈ -0.03 from the mixed-effects model; d ≈ -0.07 using animal-level aggregation), indicating minimal practical difference between groups.

**Table S5. Quantile Mixed-Effects Model Results for Motoneuron Cross-Sectional Area**

| **Model Term** | **Quartile** | **Estimate/Effect Size (µm^2^)** | **±SE** | **p-value** |
| --- | --- | --- | --- | --- |
| Control, Large Mode Cross-Sectional Area | Q1 | 1284.58 | 10.91 | p < 0.001 |
|  | Q2 | 1496.63 | 17.33 | p < 0.001 |
|  | Q3 | 1749.55 | 37.93 | p < 0.001 |
| Effect of MB on Large Mode  Cross-Sectional Area | Q1 | -49.93 | 40.99 | p = 0.224 |
|  | Q2 | -61.10 | 43.03 | p = 0.156 |
|  | Q3 | -95.53 | 53.36 | p = 0.074 |
| Large-Small Modal Difference on Control Cross Sectional Area | Q1 | -944.91 | 19.13 | p < 0.001 |
|  | Q2 | -1054.53 | 26.30 | p < 0.001 |
|  | Q3 | -1207.18 | 40.46 | p < 0.001 |
| MB Effect on Large-Small Cross-Sectional Area Difference | Q1 | +46.67 | 44.46 | p = 0.294 |
|  | Q2 | +71.73 | 43.80 | p = 0.102 |
|  | Q3 | +123.55 | 46.99 | p = 0.009 |

A quantile mixed-effects model was used to analyze the distribution of MN area across standard quartiles of both the large and small sized MN populations. The model was implemented using the R package *lqmm*, and included experimental group, size component, with animal identification as a random intercept. Alternate model structures incorporated random slope fitting for size component were evaluated to confirm model selection robustness. Model reliability was further ensured by internal centering and scaling of the MN area to improve numerical stability during fitting. Stand errors, 95% confidence intervals, and p-values were obtained via bootstrap resampling. Model terms represent effects of terms, and interactions, compared to the reference (control, large-area MNs), across equivalent quantiles. P-values reflect tests of whether mode-estimated effects differ from zero.

**Table S6. Neuromuscular Junction Postsynaptic Receptor Morphology Results**

| **Metric** | **Muscle** | **Control** | | | |  | **MB** | | | | **p-value** | **Effect Size** |
| --- | --- | --- | --- | --- | --- | --- | --- | --- | --- | --- | --- | --- |
|  |  | Mean | SD | SEM | n |  | Mean | SD | SEM | n |  |  |
| NMJ Area (µm^2^) | Extensor Digitorum Longus | 849.30 | 89.99 | 40.24 | 5 |  | 757.46 | 139.83 | 62.53 | 5 | p = 0.251 | d = 0.78 |
|  | Soleus | 973.10 | 159.03 | 60.11 | 7 |  | 895.69 | 179.12 | 73.12 | 6 | p = 0.426 | d = 0.46 |
| nAChR Area (µm^2^) | Extensor Digitorum Longus | 595.71 | 145.60 | 65.11 | 5 |  | 488.82 | 91.16 | 40.77 | 5 | p = 0.169 | d = 0.96 |
|  | Soleus | 405.87 | 81.95 | 30.97 | 7 |  | 418.89 | 76.43 | 31.20 | 6 | p = 0.561 | d = 0.33 |
| nAChR Heterogeneity | Extensor Digitorum Longus | 0.67 | 0.08 | 0.03 | 5 |  | 0.51 | 0.16 | 0.07 | 5 | p = 0.068 | d = 1.33 |
|  | Soleus | 0.53 | 0.13 | 0.05 | 7 |  | 0.51 | 0.07 | 0.03 | 6 | p = 0.796 | d = 0.14 |

Statistical comparisons between groups were performed using an independent two-sample t-test. Effect size is reported as Cohen’s d.

**Table S7. Linear Mixed Effects Model of Receptor Area as a function of NMJ Area**

| **Model Term** | **Estimate/Effect** | **95 % CI** | **±SE** | **p-value** |
| --- | --- | --- | --- | --- |
| Control, EDL nAchR Area (µm^2^) | 645.26 | [614.49, 676.03] | 15.7 | p < 0.001 |
| Control, EDL nAchR-NMJ Scaling Slope  (µm^2^/µm^2^) | 0.597 | [0.53, 0.67] | 0.036 | p < 0.001 |
| MB Effect on EDL nAchR Area (µm^2^) | -66.93 | [-111.52, -22.34] | 22.75 | p = 0.006 |
| MB Effect on EDL NMJ-nAChR Slope  (µm^2^/µm^2^) | -0.069 | [-0.17, 0.03] | 0.05 | p = 0.171 |
| EDL-SOL Difference in Control nAchR Area  (µm^2^) | -146.59 | [-178.09, -115.1] | 16.07 | p < 0.001 |
| EDL-SOL Difference in Control NMJ-nAChR Slope (µm^2^/µm^2^) | -0.154 | [-0.24, -0.07] | 0.044 | p < 0.001 |
| MB Effect on EDL-SOL nAchR Area Difference (µm^2^) | 68.31 | [22.91, 113.71] | 23.16 | p = 0.003 |
| MB Effect on EDL-SOL NMJ-nAchR Slope Difference (µm^2^/µm^2^) | 0.03 | [-0.09, 0.15] | 0.06 | p = 0.595 |

A linear mixed-effects model was used to analyze the absolute nAchR area as a function of NMJ area, muscle, and experimental group. The model was fitted using R packages *lme4* and *lmerTest*, and estimates and conclusions were compared to a robust model run in parallel using *robustlmm*. Model assumptions were assessed visually to confirm fit quality using a residual Q-Q plot, an absolute residual vs. fitted plot, and a residual of nAchR area vs. residuals of NMJ area plot. The effect estimates, 95% confidence intervals, SE, and p-values were all derived from the fitted model. Model terms are interpreted relative to the reference condition (control EDL at mean-centered NMJ area), such that all coefficients represent deviations from this baseline in either intercept or slope.

**Table S8. Model-Estimated Means from the Receptor-NMJ Area Mixed-Effects Model**

| **Metric** | **Unit** | **Muscle** | **NMJ Area** | **Control** | |  | **MB** | | **p-value** |
| --- | --- | --- | --- | --- | --- | --- | --- | --- | --- |
|  |  |  |  | Mean | 95% CI |  | Mean | 95% CI |  |
| nAchR Area | µm^2^ | Extensor Digitorum Longus | 879.12 | 645.26 | [614.49, 676.03] |  | 578.33 | [546.05, 610.60] | p = 0.007 |
|  |  | Soleus |  | 498.66 | [471.27, 526.06] |  | 500.05 | [470.95, 529.15] | p = 0.947 |
| nAchR/NMJ Slope | µm^2^/ µm^2^ | Extensor Digitorum Longus | 879.12 | 0.5974 | [0.5275, 0.6673] |  | 0.5284 | [0.4588, 0.5980] | p = 0.171 |
|  |  | Soleus |  | 0.4436 | [0.3954, 0.4918] |  | 0.4067 | [0.3640, 0.4494] | p = 0.264 |

The estimated marginal means of nAchR area and the scaling slope at the reference NMJ area, derived from the linear mixed-effects model results shown in Table S7. P-values were obtained from pairwise contrasts within each subgroup, evaluated as t-tests.

**Table S9. Mixed Model Summary of Neuromuscular Junction Morphology Metrics**

| M | Metric | Unit | Control | |  | MB | |  | P-Value | Effect Size | 95% CI | S |
| --- | --- | --- | --- | --- | --- | --- | --- | --- | --- | --- | --- | --- |
|  |  |  | EMM | 95% CI |  | EMM | 95% CI |  |  |  |  |  |
| EDL | NMJ Area | µm2 | 869.919 | [779.390, 970.964] |  | 763.313 | [683.555, 852.378] |  | p = 0.097 | 1.140 | [0.975, 1.332] | FC |
|  | nAChR Area | µm2 | 626.546 | [568.585, 684.507] |  | 519.521 | [460.904, 578.138] |  | **p = 0.012** | 0.513 | [0.118, 0.908] | FC |
|  | nAChR Fragment Area | µm2 | 606.851 | [546.869, 666.833] |  | 494.216 | [433.526, 554.905] |  | **p = 0.011** | 0.525 | [0.127, 0.922] | FC |
|  | nAChR Fragment Count | Count | 1.083 | [0.969, 1.211] |  | 1.080 | [0.969, 1.203] |  | p = 0.969† | 1.003 | [0.859, 1.171] | RR |
|  | nAChR Occupancy | % | 0.750 | [0.720, 0.777] |  | 0.709 | [0.678, 0.739] |  | p = 0.052† | 1.228 | [0.994, 1.517] | OR |
|  | nAChR Pixel Heterogeneity | % | 0.623 | [0.547, 0.693] |  | 0.497 | [0.418, 0.577] |  | **p = 0.049†** | 1.668 | [1.065, 2.610] | OR |
|  | Nerve Terminal Area | µm2 | 375.985 | [304.121, 464.830] |  | 238.300 | [188.513, 301.235] |  | **p = 0.002** | 1.578 | [1.150, 2.164] | FC |
|  | Probability of Presynaptic (+) NMJs | % | 0.966 | [0.897, 0.990] |  | 0.741 | [0.532, 0.878] |  | **p = 0.005†** | 10.086 | [2.285, 44.524] | OR |
|  | Nerve Terminal Occupancy | % | 0.390 | [0.350, 0.432] |  | 0.295 | [0.255, 0.339] |  | **p = 0.002†** | 1.529 | [1.168, 2.001] | OR |
|  | Nerve Terminal Pixel Heterogeneity | % | 0.667 | [0.590, 0.737] |  | 0.532 | [0.445, 0.618] |  | **p = 0.033†** | 1.762 | [1.085, 2.862] | OR |
|  | Nerve Terminal Area / nAChR Area | % | 0.390 | [0.350, 0.432] |  | 0.295 | [0.255, 0.339] |  | **p = 0.006†** | 1.530 | [1.169, 2.002] | OR |
| SOL | NMJ Area | µm2 | 984.286 | [895.695, 1081.640] |  | 870.034 | [785.931, 963.137] |  | p = 0.079 | 1.131 | [0.985, 1.300] | FC |
|  | nAChR Area | µm2 | 540.211 | [489.461, 590.962] |  | 505.906 | [451.284, 560.528] |  | p = 0.354 | 0.164 | [-0.193, 0.522] | FC |
|  | nAChR Fragment Area | µm2 | 404.458 | [351.867, 457.049] |  | 414.280 | [357.684, 470.877] |  | p = 0.797 | -0.046 | [-0.406, 0.314] | FC |
|  | nAChR Fragment Count | Count | 1.720 | [1.602, 1.847] |  | 1.368 | [1.255, 1.491] |  | **p < 0.001†** | 1.258 | [1.125, 1.407] | RR |
|  | nAChR Occupancy | % | 0.573 | [0.542, 0.602] |  | 0.593 | [0.561, 0.625] |  | p = 0.365† | 0.919 | [0.768, 1.099] | OR |
|  | nAChR Pixel Heterogeneity | % | 0.537 | [0.465, 0.608] |  | 0.522 | [0.444, 0.599] |  | p = 0.877† | 1.065 | [0.694, 1.632] | OR |
|  | Nerve Terminal Area | µm2 | 294.016 | [245.173, 352.590] |  | 331.171 | [271.988, 403.233] |  | p = 0.545 | 0.888 | [0.679, 1.161] | FC |
|  | Probability of Presynaptic (+) NMJs | % | 0.940 | [0.854, 0.977] |  | 0.911 | [0.789, 0.966] |  | p = 0.374† | 1.537 | [0.382, 6.178] | OR |
|  | Nerve Terminal Occupancy | % | 0.284 | [0.253, 0.316] |  | 0.341 | [0.305, 0.379] |  | **p = 0.020†** | 0.766 | [0.612, 0.959] | OR |
|  | Nerve Terminal Pixel Heterogeneity | % | 0.590 | [0.513, 0.663] |  | 0.654 | [0.574, 0.726] |  | p = 0.317† | 0.761 | [0.481, 1.205] | OR |
|  | Nerve Terminal Area / nAChR Area | % | 0.284 | [0.253, 0.316] |  | 0.341 | [0.305, 0.379] |  | **p = 0.041†** | 0.766 | [0.612, 0.958] | OR |

A comprehensive summary of all relevant neuromuscular junction morphology metrics assessed using mixed model statistics. Model estimated marginal means are shown under the EMM columns for each group, visually separated by muscle type, denoted under the Muscle (M) column. All models used a total of 460 NMJs, across two muscle types and two experimental groups, except for metrics that required presynaptic area to assess. Therefore, Nerve Terminal Area, Nerve Terminal Area / nAChR Area, Nerve Terminal Pixel Heterogeneity, and Nerve Terminal Occupancy were analyzed using 403 NMJs. Depending on distributions of data, each metric was dynamically handled for accurate statistical analysis. All normally distributed metrics were assessed with a standard linear mixed effects model, accounting for animal identification as a random intercept (using the *lmer* function of the *lme4* R package). Nonnormal distributions, including nAChR Fragment Count, Nerve Terminal Occupancy, nAChR Occupancy, Nerve Terminal Pixel Heterogeneity, nAChR Pixel Heterogeneity and Probability of Presynaptic+ NMJs were handled as either a Poisson distribution (*glmmTMB* function with family set to *compois* from the *glmmTMB* R package), beta distribution (*glmmTMB* function, with family set to *beta_family* from the *glmmTMB* R package), or binomial distribution (*glmer* function with family set to *binomial* from the *lme4* R package). P-values represent the probability of the effect size being equivalent to the null value, which is defined as 0 for normally distributed metrics, and 1 for metrics with non-normal distributions. Under the scale (S) column shows the scale of the effect size as either a fold-change (FC), odds-ratio (OR), or rate ratio (RR). The FC is a proportional fold change calculated on the natural log scale, so that an FC of 1 is equivalent to an approximately 2.718-FC across means. All p-values are derived using a two-sided t-test with the Kenward-Roger method to derive the degrees of freedom, unless denoted by (†), indicating use of the Wald z-test.
